# Enhancer Priming Enables Fast and Sustained Transcriptional Responses to Notch Signaling

**DOI:** 10.1016/j.devcel.2019.07.002

**Published:** 2019-08-19

**Authors:** Julia Falo-Sanjuan, Nicholas C. Lammers, Hernan G. Garcia, Sarah J. Bray

**Affiliations:** 1 Department of Physiology, Development and Neuroscience, University of Cambridge, Downing Street, Cambridge CB2 3DY, UK; 2 Biophysics Graduate Group, UC Berkeley, Berkeley, CA 94720, USA; 3 Department of Physics, UC Berkeley, Berkeley, CA 94720, USA; 4 Department of Molecular and Cell Biology, UC Berkeley, Berkeley, CA 94720, USA; 5 Institute for Quantitative Biosciences-QB3, UC Berkeley, Berkeley, CA 94720, USA

**Keywords:** Notch signaling, transcriptional bursting, burst duration, enhancer, enhancer priming, live imaging, *Drosophila*

## Abstract

Information from developmental signaling pathways must be accurately decoded to generate transcriptional outcomes. In the case of Notch, the intracellular domain (NICD) transduces the signal directly to the nucleus. How enhancers decipher NICD in the real time of developmental decisions is not known. Using the MS2-MCP system to visualize nascent transcripts in single cells in *Drosophila* embryos, we reveal how two target enhancers read Notch activity to produce synchronized and sustained profiles of transcription. By manipulating the levels of NICD and altering specific motifs within the enhancers, we uncover two key principles. First, increased NICD levels alter transcription by increasing duration rather than frequency of transcriptional bursts. Second, priming of enhancers by tissue-specific transcription factors is required for NICD to confer synchronized and sustained activity; in their absence, transcription is stochastic and bursty. The dynamic response of an individual enhancer to NICD thus differs depending on the cellular context.

## Highlights

• Notch-responsive enhancers are activated synchronously in *Drosophila* embryos
• Notch levels modulate transcription by changing burst size
• Notch elicits stochastic versus deterministic activation depending on cell context
• Priming by Twist and Dorsal promotes fast and sustained response to Notch

## Graphical Abstract

**Figure:**
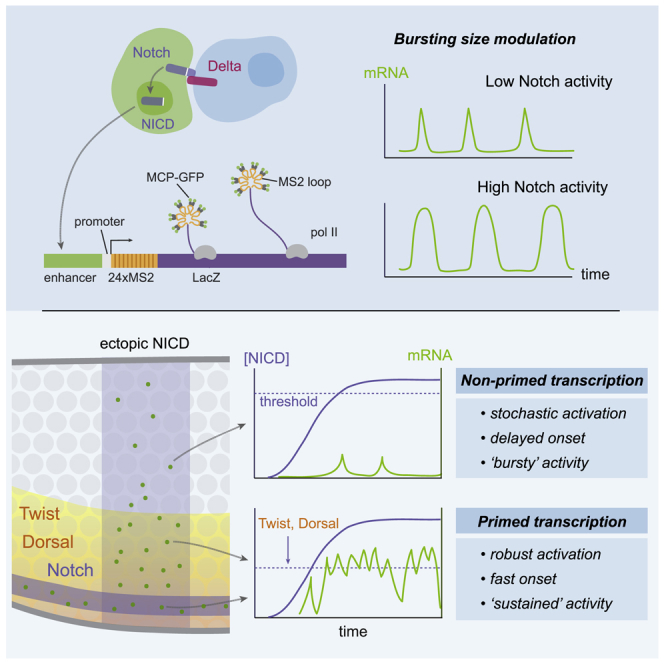

## Teaser

Falo-Sanjuan et al. use live imaging to investigate how enhancers detect and respond to Notch signaling in real time *in vivo*. They discover that responses are synchronized and sustained in the normal context, due to local transcription factor priming, and that elevated Notch activity increases transcription burst size rather than frequency.

## Introduction

Genes respond to external and internal cues through the actions of transcription factors and effectors of signaling pathways. Gene regulatory regions, termed enhancers, integrate information from these inputs to produce an appropriate transcriptional output. During development, decisions may occur in a matter of minutes, but as the transcription dynamics have rarely been analyzed *in vivo* in real time, we know little about how recipient enhancers decipher the signals. For example, enhancers could respond in a digital manner, working as simple on-off switches, or as analog devices, operating as a rheostat so that signal levels can modulate the output (Blackwood and Kadonaga, 1998, Garcia et al., 2013, Lammers et al., 2018). In either case, they must also have the capability to detect and transduce key parameters to the transcription machinery, such as signal duration and thresholds.

With the advent of precise and quantitative methods to measure transcription, such as single molecule fluorescence *in situ* hybridization (smFISH) or live imaging, it has become evident that transcription is not a continuous process. Instead, transcribing genes undergo bursts of initiation that are often separated by inactive intervals (Chubb et al., 2006, Golding et al., 2005). Bursting is thought to occur because dynamic enhancer-promoter activation leads to episodic polymerase release. One consequence of this is that factors modulating the levels of transcription can do so by changing either the frequency with which a burst occurs (measured by the gap between bursts) or the size of each burst (measured by changes in burst duration and/or amplitude). To date, bursting frequency rather than burst duration or amplitudes seems to be the major parameter modulated in different species and contexts (So et al., 2011, Senecal et al., 2014, Xu et al., 2015, Desponds et al., 2016, Padovan-Merhar et al., 2015, Lammers et al., 2018, Berrocal et al., 2018). For example, enhancers controlling early patterning genes in *Drosophila* embryos produce similar bursting size but have different bursting frequencies, which can be attenuated by the presence of insulators (Fukaya et al., 2016). Similarly, steroids increase the bursting frequency of target enhancers (Larson et al., 2013, Fritzsch et al., 2018). However, it remains to be discovered whether all transcription factors alter transcription dynamics in this way and specifically whether it is these or other properties that are modulated by developmental signals to confer appropriate outputs in the *in vivo* setting of a developing organism.

Transcriptional bursting is thought to make an important contribution to heterogeneity in transcriptional activity between cells (Raj and van Oudenaarden, 2008). For example, in cells exposed to estrogen, response times for transcription activation were highly variable with no coherent cycling between active and inactive states (Fritzsch et al., 2018). Stochastic transcriptional behavior is also of key importance for differentiation of photoreceptors in *Drosophila* eyes (Wernet et al., 2006), of hematopoietic cells in mice (Chang et al., 2008, Ng et al., 2018), and of neuronal cells in zebrafish retina (Boije et al., 2015). However, while it is an attractive feature for promoting heterogeneity, inherent transcriptional variability could be extremely disruptive in developmental processes where a coordinated response of many cells is required to pattern specific structures. In some cases, this may be circumvented by averaging mechanisms that allow cells to produce homogeneous patterns of gene expression (Little et al., 2013) that include mRNA diffusion in *Drosophila* syncytial embryos (Bothma et al., 2018). However, it is only in rare circumstances that mRNA diffusion can operate, and it is unclear whether other averaging mechanisms would be effective over shorter time intervals. To effectively achieve reproducible patterns, cells must therefore overcome the variability that is inherent in transcriptional bursting and stochastic enhancer activation.

Notch signaling is a highly conserved developmental signaling pathway that is deployed in multiple contexts. It has the unusual feature that the Notch intracellular domain (NICD) transduces the signal directly to the nucleus, when it is released by a series of proteolytic cleavages precipitated by interactions with the ligands. NICD then stimulates transcription by forming a complex with the DNA binding protein CSL and the co-activator Mastermind (Mam) (Bray, 2006). The lack of intermediate signaling steps and amplification makes this a powerful system to investigate how signals are deciphered by responding enhancers. Furthermore, there may be differences in the levels and dynamics of NICD produced by different ligands (Nandagopal et al., 2018). However, although its role as a transcriptional activator is well established, at present, we know little about how enhancers respond to NICD in the real time of developmental decisions. For example, do enhancers operate as simple switches, detecting when NICD crosses a threshold, or are they sensitive to different levels of NICD, in which case does NICD, like other factors, modulate bursting frequency? Nor do we know what sequence features in the responding enhancers confer the output properties, although enhancers with paired CSL motifs (referred to as SPS motifs) (Bailey and Posakony, 1995, Nam et al., 2007), whose precise spacing could favor NICD dimerization, are suggested to yield the strongest responses (Nam et al., 2007).

In order to determine how enhancers respond to Notch activity in real time, we have used the MS2-MCP system to visualize nascent transcripts in *Drosophila* embryos. To do so, we used two well-characterized Notch-responsive enhancers that drive expression in a stripe of mesectoderm (MSE) cells and analyzed their transcription profile over time at the single cell level. Strikingly, all MSE cells initiated transcription within a few minutes of one another, and once active, each nucleus produced a sustained profile of transcription. By manipulating NICD levels and altering key motifs within the enhancers, we uncover two key principles. First, the ability of NICD to confer synchronized and sustained activity in MSE requires that the enhancers be primed by tissue-specific transcription factors. In their absence, MSE enhancers confer stochastic bursty transcription profiles, demonstrating that different response profiles can be generated from a single enhancer according to which other factors are present. Second, changing Notch levels modulate the transcription burst size but not the inter-burst periods, in contrast to most current examples of enhancer activation. These two key concepts are likely to be of general importance for gene regulation by other signaling pathways in developmental and disease contexts.

## Results

### Synchronized and Sustained Enhancer Activation in Response to Notch

**Figure 1:**
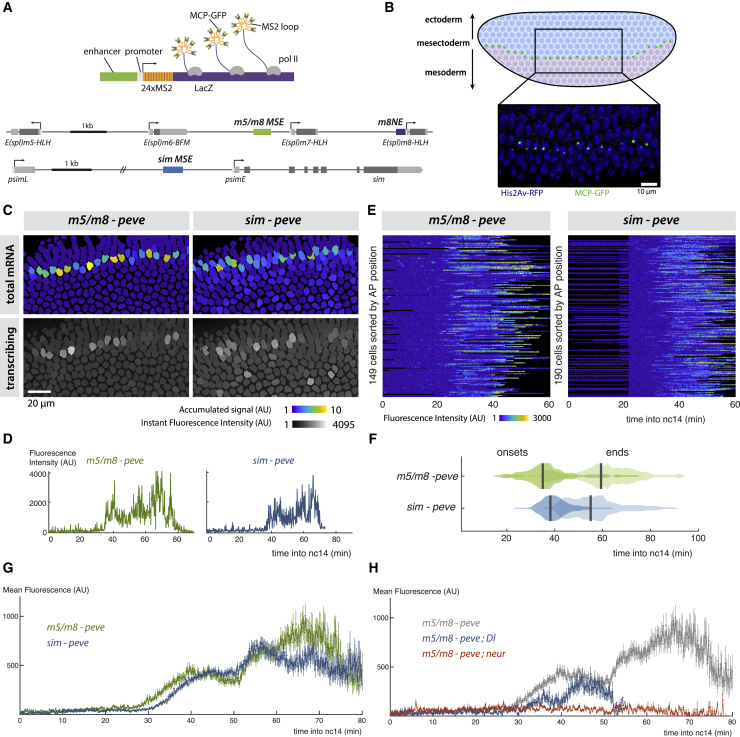
Synchronous Activity of Two Notch-Responsive Enhancers (A) Diagrams illustrating the MS2 strategy for live imaging of transcription (top) and the location of mesectoderm (MSE) and neuroectoderm (NE) enhancers in *E(spl)-C* (*m5/m8* ; *m8NE*) and *single minded* (*sim*) (bottom). Arrows indicate transcription start-sites, boxes in lower panel indicate promoters (white), non-coding (light gray) and coding (dark gray) transcribed regions. (B) Diagram of a blastoderm *Drosophila* embryo, indicating mesodermal Delta expression (pink), which activates Notch in flanking cells (green dots) to specify the MSE. Image: transcription from *m5/m8* detected by MCP-GFP accumulation in bright puncta (green), nuclei are labeled by His2Av-RFP (blue). (C) Tracked expression from *m5/m8* and *sim* reporters. Top panels: tracked nuclei false-colored by total signal levels, proportional to total mRNA production. Bottom panels: single frames with tracked nuclei shaded according to maximum pixel intensity. (D) Profiles of *m5/m8* and *sim* fluorescence from individual nuclei that exhibit “sustained” activity. (E) Heatmaps representing fluorescence profiles of *m5/m8* and *sim* in all MSE nuclei during nc14 (scale as indicated with blue, no expression; yellow, high expression; black indicates periods where nuclei were not tracked). (F) Distributions of onsets and end points of transcription from *m5/m8* and *sim* in the MSE. (G) *m5/m8* and *sim* produce similar average temporal profiles. Mean fluorescent intensity of MCP-GFP puncta at indicated times in nc14. (H) Transcription from *m5/m8* is curtailed in embryos lacking zygotic Delta (Dl) and abolished in embryos lacking *neuralized* (neur). Gray trace, *m5/m8* profile in wild-type embryos from (G). In (G) and (H), mean and SEM of all MSE cells are shown. n = 3 (*m5/m8*), 3 (*sim*), 2 (*m5/m8 ; Dl*), 2 (*m5/m8 ; neur*) embryos. In this and other figures, the *peve* promoter was used in all reporters unless otherwise specified. See also Figure S1 and Videos S1 and S2.

To investigate how Notch signals are read out by an enhancer in real time, we focused on well-characterized MSE enhancers from the *Enhancer of split-Complex (E(spl)-C)* (known as *m5/m8*) and from *singleminded* (*sim*) (Morel and Schweisguth, 2000, Cowden and Levine, 2002, Zinzen et al., 2006a). These direct expression in two stripes of MSE cells during nuclear cycle 14 (nc14) when Notch is activated in response to Delta signals from the presumptive mesoderm (ME) (Figures 1A and 1B) (Morel et al., 2003, De Renzis et al., 2006, Zinzen et al., 2006a). The MSE converges to the midline during gastrulation, ultimately forming CNS midline precursors similar to the vertebrate floorplate. To visualize transcription from MSE enhancers in real time and define the response properties conferred by a defined enhancer DNA sequence, they were inserted into MS2 reporter constructs comprising the even-skipped promoter (*peve*), 24 *MS2* loops, and *lacZ* (Figure 1A). When combined with MCP-GFP in the same embryos, nascent transcription from the MS2 reporters was detected by the accumulation of MCP-GFP in nuclear puncta, whose fluorescence is directly proportional to the number of transcribing mRNAs at any time point (Figures 1A and 1B) (Garcia et al., 2013). In this way, levels of transcription can be followed over time in each cell by tracking the puncta relative to nuclei.

Visualizing transcription in real time revealed that *m5/m8* and *sim* were both activated in all MSE cells within a narrow time window (∼10 min) in nc14 (Figures 1C, 1E, and 1F; Videos S1 and S2). Activity was then maintained in these nuclei throughout the remaining period of nc14 as embryos underwent gastrulation. Both *m5/m8* and *sim* exhibited what we refer to as “sustained activity” because each punctum retained high levels of fluorescence rather than exhibiting clearly distinct bursts (Figure 1D), although we note that the resolution of bursting events is limited by the time each polymerase takes to complete transcription (estimated as 1.6–2.5 min for these reporters) (Fukaya et al., 2017). Transcription then ceased after 30–50 min, with less synchrony than at the onset (Figure 1F). Identical response profiles were obtained when the *m5/m8* reporter was inserted at a different genetic locus (Figures S1A and S1B).

Video S1. Expression of *m5/m8-peve*, Related to Figure 1 Video showing transcription from *m5/m8-peve* in the mesectoderm stripe during nc14. Also note earlier, Notch-independent, transcription in broad domains in nc10-13 and in few scattered cells during the first few minutes of nc14, followed by a long period (approximately 20 min) of inactivity before MSE stripe cells initiate transcription. Maximum intensity projection (19x1μm stacks) of the MCP-GFP (gray in left panel, green in right panel) and His2Av-RFP (blue in right panel) channels. 0.36 μm/px XY resolution and final time resolution of 10s/frame. Anterior to the left; embryo imaged from the ventral side.

Video S2. Expression of *sim-peve*, Related to Figure 1 Video showing transcription from *sim-peve* in the mesectoderm stripe and some mesodermal cells during nc14. Also note activity in scattered cells before nc14. Maximum intensity projection (29x1μm stacks) of the MCP-GFP (gray in left panel, green in right panel) and His2Av-RFP (blue in right panel) channels. 0.36 μm/px XY resolution and final time resolution of 15s/frame. Anterior to the left; embryo imaged from the ventral side.

Sustained activity is a feature of *m5/m8* and *sim* and not a general property of Notch-responsive enhancers at this stage, as a neurectodermal enhancer from *E(spl)m8-bHLH* (*m8NE*, Figure 1A) produced profiles where individual bursts of activity were clearly resolved, which we refer to as “bursty” (Figures S1A–S1C). Furthermore, even though profiles produced by *m5/m8* and *sim* were continuous, their amplitude fluctuated, likely reflecting episodic polymerase release. Overall, however, the *m5/m8* and *sim* response profiles were highly coordinated temporally (Figures 1E and 1F). Indeed, the mean profile of all MSE cells analyzed was almost identical for the two enhancers (Figure 1G). This is remarkable given that they contain different configurations of binding motifs and implies that MSE cells undergo a highly synchronized period and level of Notch signaling.

To assess the relative contributions of the enhancer and promoter to response profiles, we next tested consequences of substituting different promoters with *m5/m8* and *sim*, inserting the reporters at the same genomic position to ensure comparability. First, when *peve* was replaced by a promoter from *sim* (*psimE*), both *m5/m8* and *sim* produced lower levels of transcription, but their overall temporal profiles remained similar (Figure S1D). Second, when we combined *m5/m8* with another heterologous promoter, *hsp70*, or with four promoters from the *E(spl)-C* locus, mean levels of transcription were again affected without changing the overall temporal profile or expression pattern (Figure S1E). Notably, even in combinations yielding lower levels, e.g., *pm6* (Figure S1E), the transcription profiles remained sustained rather than breaking down into discrete bursts (Figure S1F). Although the results suggest there could be an underlying enhancer-promoter compatibility at the sequence level (Figure S1E) (Zabidi et al., 2015), there was no obvious relationship between the mean levels of transcription produced by a promoter and the presence or absence of sequence motifs for factors associated with promoter accessibility, such as Zelda or Trithorax-like (Blythe and Wieschaus, 2016). Nor was there a correlation between promoter activity with *m5/m8* in the MSE and that with a heterologous developmental enhancer in *Drosophila* S2 cells (Arnold et al., 2016). However, since the promoter substitutions had no effect on temporal profiles, it argues that the enhancers are the primary detectors of Notch activity.

To verify that MSE transcription was Notch dependent, we measured transcription from *m5/m8* in embryos where Notch activity was disrupted by mutations. Embryos lacking Neuralized, an E3 ubiquitin ligase required for Delta endocytosis that is critical for Notch signaling (Morel et al., 2003, De Renzis et al., 2006), had no detectable transcription from *m5/m8* in the MSE (Figure 1H). Likewise, *m5/m8* activity was severely compromised in embryos carrying mutations in *Delta*. Because Delta protein is deposited in the egg maternally (Kopczynski et al., 1988), these embryos contained some residual Delta, which was sufficient for a few scattered MSE cells to initiate transcription (Figure S1G). However, their transcription ceased prematurely, within <20 min (Figures 1H and S1G). Together, these results confirm that the enhancers require Notch signaling for their activity in the MSE, in agreement with previous studies (Morel and Schweisguth, 2000, Zinzen et al., 2006a), and further show that continued Notch signaling is needed to maintain transcription, arguing that the MSE enhancers also detect persistence of NICD.

### Coordinated Activity of Enhancers within Each Nucleus

**Figure 2:**
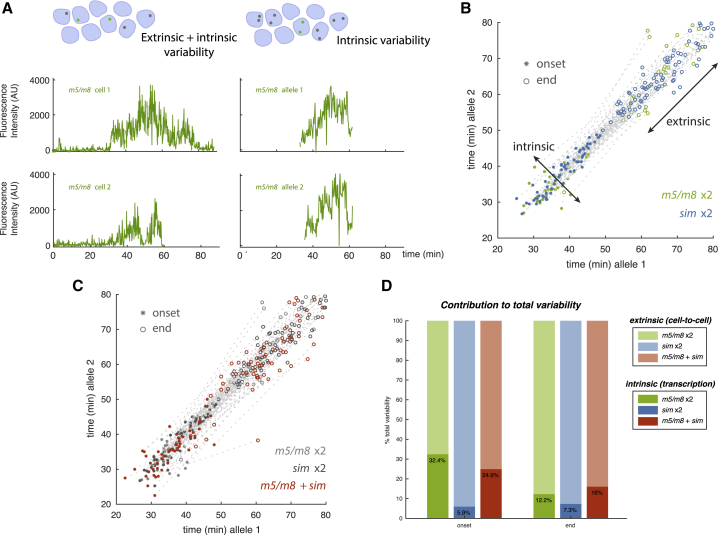
Notch Enhancers Exhibit Low Intrinsic Variability (A) Examples of transcription profiles from *m5/m8* in different nuclei (left panels) and from two alleles in the same nucleus (right panels). (B) Onset and end points of activity from individual punctum in nuclei with two *m5/m8* or *sim* alleles. Distribution across the diagonal, intrinsic variability (within cells); distribution along the diagonal, extrinsic variability (between cells). (C) Onset and end points of activity from individual punctum in nuclei carrying an *m5/m8* allele and a *sim* allele (data from individual enhancers, C, gray, for comparison). (D) Contribution of intrinsic variability (dark shading) to variability in transcription onset and end times in the indicated two-allele combinations. Connecting gray lines indicates onset and end times from the same nucleus. n = 2 (*m5/m8* × 2), 3 (*sim* × 2), 3 (m5/m8 + *sim*) embryos. See also Figure S2.

Although *m5/m8* and *sim* confer well-coordinated temporal transcription profiles, their precise time of activation shows some cell-to-cell variability (Figure 1F). To investigate whether this variability reflects stochastic variations in transcription (intrinsic variability) or differences in signaling from Notch (extrinsic variability) (Elowitz, 2002, Raser and Shea, 2006), we monitored expression from two identical alleles of the MS2 reporters (Figure 2A). Transcription from these two physically unlinked loci was detected as distinct puncta in each nucleus, which could be tracked independently. We found a remarkable synchrony in the onset of transcription from both alleles of a given enhancer (Figure 2B). More than 80% of cells initiated transcription from both alleles within 5 min (Figure S2C). This contributes to ∼6%–30% of the total variability (Figure 2D), indicating that most onset variability was due to extrinsic factors. Transcription was extinguished less synchronously (Figures 2B and S2A), but this intrinsic variability was still much less than that between cells (Figure 2D).

Although two alleles in the same cell gave overall similar profiles, their fine-grained spikes and troughs were not synchronized (Figure 2A), as expected if transcription from two different loci is largely uncorrelated (Harper et al., 2011, Little et al., 2013, Fritzsch et al., 2018). However, their fluorescence intensities displayed a small but significant positive correlation (R^2^ ∼ 0.35) (Figure S2B). This argues that the enhancers at the two alleles operate independently while being coordinated by the same extrinsic information. Strikingly, when *m5/m8* and *sim* were present in *trans* in the same cell, there was also comparatively little variation in their onset times (Figures 2C, 2D, and S2A). Thus, the properties of *m5/m8* and *sim* ensure that they reliably detect extrinsic information in the form of Notch activity, initiated within a 5- to 10-min time window so that their activation is remarkably synchronized within each nucleus.

### Enhancers Detect Signal Thresholds and Context

**Figure 3:**
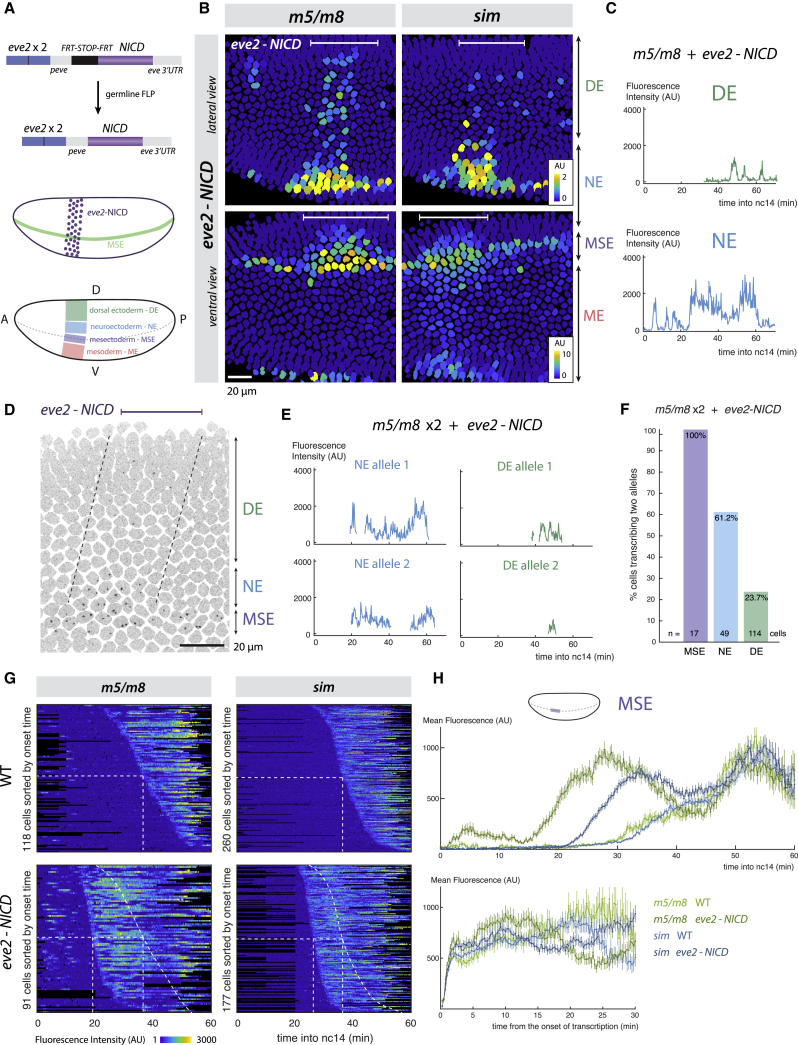
Effects of Ectopic NICD on Temporal Transcription Profiles Reveal that Enhancers Have Different Thresholds (A) Strategy for producing ectopic NICD using *eve2*, with schematics depicting expression (purple shading) relative to MSE (green) and DV regions where effects on transcription were quantified. (B) Still frames of tracked nuclei false-colored for total accumulated signal (note different scales). DE, NE, MSE, and ME correspond to the regions shown in (A). (C) Illustrative traces from DE (top) and NE (bottom) nuclei, where NICD elicits different *m5/m8* transcription profiles. (D) Still frame, *eve2-NICD* embryo with two *m5/m8* alleles. Inverted maximum intensity projection of MCP-GFP is overlaid with outlines of tracked nuclei, dashed lines indicate region of ectopic NICD. (E) Examples of transcription traces from two *m5/m8* alleles in NE or DE nuclei. (F) Proportion of nuclei that ever transcribe two *m5/m8* alleles at the same time. (G) Heatmaps of transcription traces from *m5/m8* and *sim* in MSE nuclei from wild-type and *eve2-NICD* embryos, sorted by onset time. Dashed lines indicate onset times in wild-type embryos. (H) Mean activity profiles in MSE nuclei over time (top) and aligned by onset time (bottom; transcription in each nucleus increases steeply in all conditions). H mean and SEM of all MSE cells. n = 4 (*m5/m8* WT), 7 (*sim* WT), 6 (*m5/m8 eve2-NICD*), 8 (*sim eve2-NICD*), 4 (*m5/m8* ×2 *eve2-NICD*) embryos. See also Videos S3, S4, S5, and S6.

The *m5/m8* and *sim* enhancers appear to act as “persistence detectors,” driving transcription as long as Notch signal(s) are present. They may simply detect when Notch reaches a threshold (digital encoding) or they could be sensitive to Notch activity levels (analog encoding). To distinguish these possibilities, we supplied ectopic NICD using the *stripe 2* regulatory enhancer from *even-skipped* (*eve2-NICD*). This produces an ectopic stripe of NICD, orthogonal to the MSE (Figure 3A) (Kosman and Small, 1997, Cowden and Levine, 2002), which was sufficient to produce ectopic expression from both *m5/m8* and *sim* (Videos S3 and S4).

Video S3. Ectopic Expression of *m5/m8* with *eve2-NICD*, Related to Figure 3 Video showing ectopic tran- scription from *m5/m8-peve* in the *eve2* domain during nc14. Maximum intensity projection (29x1μm stacks) of the MCP-GFP (gray in left panel, green in right panel) and His2Av-RFP (blue in right panel) channels. 0.36 μm/px XY resolution and final time resolution of 15s/frame. Anterior to the left; embryo imaged from the ventral side.

Video S4. Ectopic Expression of *sim* with *eve2-NICD*, Related to Figure 3 Video showing ectopic transcription from *sim-peve* in the *eve2* domain during nc14. Maximum intensity projection (29x1μm stacks) of the MCP-GFP (gray in left panel, green in right panel) and His2Av-RFP (blue in right panel) channels. 0.36 μm /px XY resolution and final time resolution of 15s/frame. Anterior to the left; embryo imaged from the ventral side.

Whereas expression from *m5/m8* and *sim* was almost identical in wild-type embryos, clear differences were revealed by ectopic NICD. First, *m5/m8* was activated throughout the dorsal *eve2* domain whereas *sim* only responded in a 3- to 4-cell region close to the MSE (Figure 3B), consistent with previous observations (Cowden and Levine, 2002, Zinzen et al., 2006a). Second, although both enhancers initiated transcription prematurely because the ectopic NICD was produced from early nc14 (Bothma et al., 2014), *m5/m8* was switched on significantly earlier than *sim* (Figures 3G and 3H). Given that both enhancers are exposed to the same temporal pattern of NICD, this difference in initiation times implies that they respond to different thresholds of NICD. Therefore, we hypothesize that *m5/m8* and *sim* respond at the same time in wild-type embryos because the normal ligand-induced signaling leads to a sharp increase in NICD.

We also detected differences in the response dynamics of *m5/m8* according to location. Nuclei close to the MSE stripe (in the neuroectoderm, NE) exhibited strong activity, with a temporal pattern resembling that in the MSE (Figure 3C, bottom). In contrast, nuclei in more dorsal regions (dorsal ectoderm, DE) underwent resolved bursts of transcriptional activity (Figure 3C, top). Ectopic NICD also induced “bursty” expression from *sim* in the ME but was not capable of turning on *m5/m8* in that region (Video S5).

Video S5. Regions of Ectopic Expression of *m5/m8* and *sim* with *eve2-NICD*, Related to Figure 3 Combined video of *m5/m8-peve* (left) and *sim-peve* (right) showing ectopic transcription in the *eve2* stripe. The maximum projection of the MCP-GFP signal is overlaid with tracked nuclei false colored with the maximum intensity pixel in each nucleus. Active nuclei in each of the analyzed regions are marked with a different color: red (mesoderm), purple (mesectoderm), blue (neuroectoderm) and green (dorsal ectoderm). Anterior to the left; embryo imaged from the ventral side.

“Bursty” *m5/m8* transcription in the DE was also associated with more stochastic activation. In embryos with two *m5/m8* alleles, both were activated in response to *eve2-NICD* in most MSE and NE nuclei, whereas only a single allele was active at any one time in most DE nuclei (Figures 3D and 3F; Video S6). Furthermore, in the few DE nuclei where both alleles became active, there was greater variability in onset times and the profiles were less coordinated (Figures 3E and S2D). The positional differences in dynamics suggest that intrinsic cellular conditions, likely expression levels of specific transcription factors, influence the way that enhancers “read” the presence of NICD. Such factors must therefore have the capability to modulate the dynamics of transcription.

Video S6. Transcription of Two *m5/m8-peve* MS2 Reporters in the Presence of Ectopic NICD, Related to Figure 3 Video showing ectopic transcription from *m5/m8-peve* x2 in the *eve2* domain during nc14. Maximum intensity projection (29x1μm stacks) of the MCP-GFP (gray in left panel, green in right panel) and His2Av-RFP (blue in right panel) channels. 0.36 μm /px XY resolution and final time resolution of 15s/frame. Anterior to the left; embryo imaged from the ventral side.

### Notch Activity Tunes Transcription Burst Size

**Figure 4:**
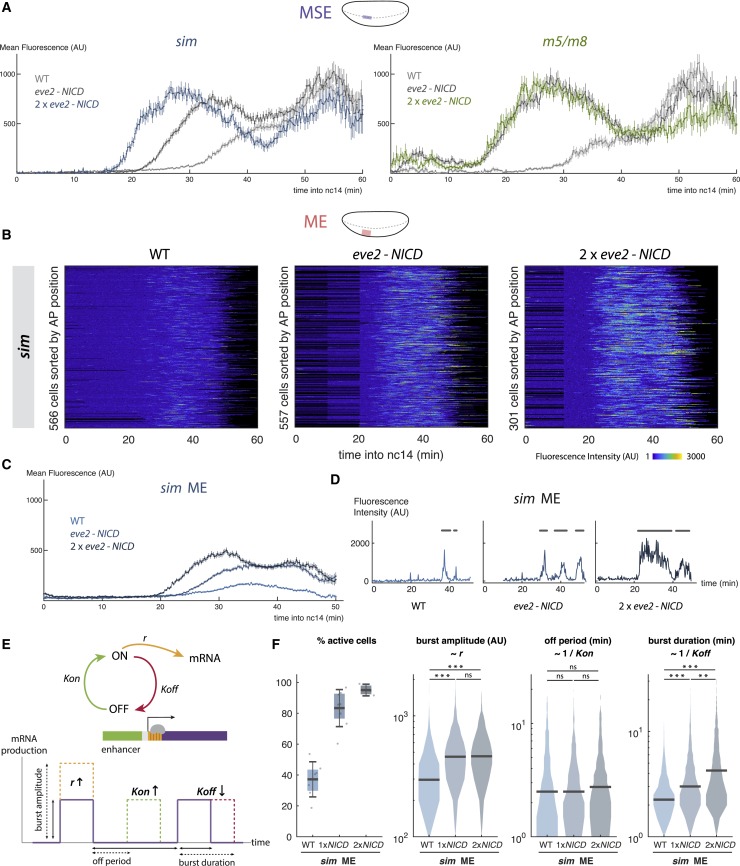
Notch Produces a Dose-Sensitive Response by Regulating Transcription Burst Size (A) Mean levels of transcription from *sim* (left) and *m5/m8* (right) in the MSE with (2x*eve2-NICD*), compared to 1x*eve2-NICD* and to the wild type. (B) Heatmaps depicting *sim* activity in ME nuclei in three conditions as indicated. Note the different scale range compared to Figure 3G. (C) Mean levels of transcription from *sim* in ME produced by different doses of NICD. (D) Examples of transcription traces from single ME nuclei in the wild type, 1x*eve2-NICD*, and 2x*eve2-NICD*. Burst periods are marked with a gray line. (E) Schematic of the model: an enhancer cycles between ON and OFF states and produces mRNA when ON. Changes in bursting amplitude, off period, and bursting duration correlate with changes in kinetic constants *r*, *K*_*on*_, and *K*_*off*_. (F) Quantification of individual burst properties from *sim* in ME of the wild type, 1x*eve2-NICD*, and 2x*eve2-NICD* embryos. Boxplots indicate median, with 25–75 quartiles; error bars are SD. Violin plots, distributions of the analyzed bursts, bar indicates the median. In (A) and (C), mean fluorescence values and SEM are plotted. Gray lines are reproduced from Figure 3H. n cells for (B)–(F) are indicated in (B). Differential distributions tested with two-sample Kolmogorov-Smirnov test: p values < 0.01(^∗^), < 10^−5^ (^∗∗^), <10^−10^ (^∗∗∗^). n = 3 (*m5/m8* 2x*eve2-NICD*), 3 (*sim* 2x*eve2-NICD*) embryos. See also Figures S3 and S4.

To further test how Notch-responsive enhancers respond to doses of signal, we introduced a second *eve2-NICD* transgene. MSE transcription from *sim* in 2x*eve2-NICD* embryos initiated earlier and achieved higher levels than with 1x*eve2-NICD* (Figure 4A, left). This agrees with the hypothesis that *sim* responds to higher thresholds of NICD, as nuclei will reach a given concentration of signal more quickly in embryos with 2x*eve2-NICD*. The mean levels of transcription increased in ME as well as in MSE regions (Figures 4A–4C), further indicating a dose-sensitive response. In contrast, levels and onset of MSE transcription from *m5/m8* did not significantly change in 2x*eve2-NICD* embryos (Figure 4A, right). This saturation in output from *m5/m8* only occurred in the MSE, as the more stochastic activity in the DE remained sensitive to increases in NICD, being detected in a greater proportion of cells and over longer periods (Figure S4A).

To distinguish different models for how NICD confers a dose-sensitive response, we took two strategies to analyze its effect on transcriptional bursting dynamics and focused on regions where individual bursts of transcription were resolved. Both approaches assume a two-state model where the enhancer is switched between an OFF and ON state with switching rates *K*_*on*_ and *K*_*off*_ and confers transcription initiation rate *r* in the ON state (Figure 4E) (Peccoud and Ycart, 1995, Larson et al., 2009). In the first approach, we directly measured bursting amplitude, off period between bursts and bursting length as approximations for *r*, *K*_*on*_, and *K*_*off*_, respectively (Figure 4E). In most previous enhancers analyzed in this way, the off period is the most affected, leading to changes in the bursting frequency (Fukaya et al., 2016, Fritzsch et al., 2018, Lammers et al., 2018). However, when we quantified the effect from different doses of NICD on *sim* in the ME, we found that bursting length consistently increased with higher amounts of NICD, whereas off periods between bursts remained constant (Figures 4D and 4F). This indicates that the main effect of NICD is to keep the enhancer in the ON state for longer—i.e., decreasing *K*_*off*_—rather than increasing the frequency with which it becomes active—i.e., increasing *K*_*on*_. The bursting amplitude also increased with 1x*eve2-NICD* but this was not further enhanced by 2x*eve2-NICD* (Figures 4D and 4F). Overall, therefore, increasing levels of NICD in the ME result in *sim* producing an increase in transcription burst size (duration × amplitude) rather than an increase in the frequency of bursts. A similar increase in burst size in response to the dose of NICD (Figures S4A–S4C) occurred with other regions and enhancers (*m5/m8* DE and *m8NE* ME), suggesting that it is a general property of these Notch-responsive enhancers.

We developed a second approach, based on the noise properties of transcription, to analyze the changes in dynamics even where single bursts of activity could not be defined. To do so, we used a mathematical model of transcription to account for the initiating mRNA molecules (Figure S3A). Using derivations from the mathematical model and testing them in simulations, we looked for signatures that would be produced if the mean of initiating mRNAs (equivalent to the mean fluorescence from MS2 puncta) were increasing due to changes in *r*, *K*_*on*_, or *K*_*off*_. This showed that the effects on the Fano factor ratio between the two conditions and on their autocorrelation function (ACF) could be used to correctly predict which of the parameters could account for the increase (Figure S3B; STAR Methods). First, we tested the modeling approach with the data from the promoter swap experiments. Analyzing the differences in the mean indicated that they are most likely due to increases in *r* (Figure S4D), as expected if promoters influence the rate of polymerase release but not enhancer activation per se. When we then applied the model to data from the transcription profiles produced by different doses of NICD in the ME, results were most compatible with the causal effect being an increase in *r* or a decrease in *K*_*off*_ (Figure S4E) depending on which two conditions were compared. Thus, this second approach also indicated that NICD elicits an increase in burst size rather than in burst frequency. Both approaches therefore converge on the model that above the critical threshold level of NICD, further increases in NICD levels prolong the period that each enhancer remains in the ON state.

Finally, we used an enhancer-promoter combination that produced higher mean levels (*m5/m8-pm5*, Figure S1E) to investigate whether the saturation that occurred with ectopic NICD was due to the *peve* promoter having achieved maximal initiation rate. Strikingly, the substitution of *pm5* for *peve* did not result in significantly higher maximal levels in the presence of *eve2-NICD* (Figure S4F), although it did in wild-type embryos (Figure S1E). This indicates that the saturation of the response with higher levels of NICD stems from the *m5/m8* enhancer rather than the promoter and argues that enhancers reach a maximal “ON” state that they cannot exceed even if more NICD is provided.

### Paired CSL Motifs Augment Burst Size, Not Threshold Detection

**Figure 5:**
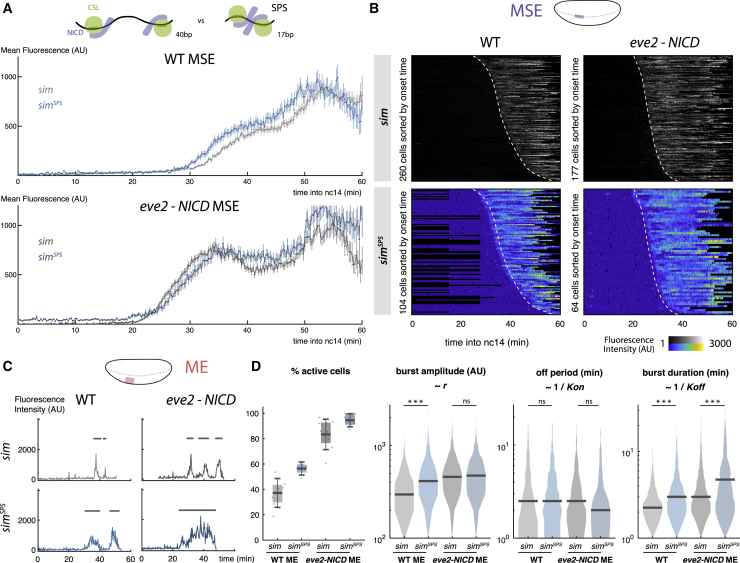
Optimized Su(H) Motif Organization Enhances Bursting Size (A) Mean levels of transcription in MSE nuclei when two Su(H) motifs in *sim* are replaced with an optimal paired SPS motif (*sim*^*SPS*^) in wild-type (top) and *eve2-NICD* (bottom) embryos. Mean and SEM shown. (B) Heatmaps of transcription in MSE nuclei from *sim*^*SPS*^ and *sim* in wild-type and 1x*eve2-NICD* embryos, sorted by onset time. Dashed lines indicate onset times for unmutated *sim*. (C) Examples of fluorescent traces from *sim* and *sim*^*SPS*^ in ME nuclei. Burst periods are indicated by gray lines. (D) *sim*^*SPS*^ activity compared to *sim*. Boxplots indicate median, 25–75 quartiles, and error bars are SD. Violin plots, distribution for all bursts measured in the ME, bar indicates the median. Differential distributions tested with two-sample Kolmogorov-Smirnov test: p values < 0.01(^∗^), < 10^−5^ (^∗∗^), < 10^−10^ (^∗∗∗^). n = 4 (*sim*^*SPS*^ WT) and 6 (*sim*^*SPS*^*eve2-NICD*) embryos. Gray lines, heatmaps, and violin plots are re-plotted from Figures 3G, 3H, 4D, and 4F for comparison. See also Figure S5.

*m5/m8* and *sim* enhancers both respond to NICD but they initiate transcription at different thresholds. How is this encoded in their DNA sequence? A prominent difference is that *m5/m8* contains a paired CSL motif (so-called SPS motif) whereas *sim* does not (Figure S5A). To test their role, we replaced two CSL motifs in *sim* with the SPS motif from *m5/m8* and conversely perturbed the SPS in *m5/m8* by increasing the spacing between the two CSL motifs (Figure S5A). As SPS motifs permit cooperative binding between two NICD complexes, we expected that enhancers containing an SPS motif (*sim*^*SPS*^ and *m5/m8*) would exhibit earlier onsets of activity than their cognates without (*sim* and *m5/m8*^*insSPS*^). However, this was not the case for either *sim* and *sim*^*SPS*^ (Figures 5A and 5B) or *m5/m8* and *m5/m8*^*insSPS*^ in either wild-type or *eve2-NICD* embryos (Figures S5D and S5E). These profiles suggest that the SPS motifs are not responsible for the difference in the threshold levels of NICD required for *m5/m8* and *sim* activation.

Changes to the CSL motifs did, however, affect mean levels of activity. *sim*^*SPS*^ directed higher mean levels of activity compared to *sim* in both wild-type and *eve-NICD* embryos (Figures 5A and S5B). Conversely, *m5/m8*^*insSPS*^ directed lower levels compared to *m5/m8* (Figure S5D). Analyzing the traces from *sim* in the ME, where cells undergo resolved bursts of transcription, revealed that the SPS motif (*sim*^*SPS*^) led to larger burst sizes—i.e., increased the amplitude and the duration—compared to wild-type *sim* (Figures 5C and 5D). Conversely, the continuous profile produced by *m5/m8* in the MSE was broken into smaller bursts when the SPS was disrupted (Figures S5F and S5G). The effects on bursting size are similar to those seen when the dose of NICD was altered, suggesting that enhancers containing SPS sites respond to a given level of NICD more effectively. They do not, however, appear to affect the amount of NICD required for their initial activation, i.e., the threshold required for the enhancer to be switched on. This implies that burst size modulation and response threshold can be uncoupled and potentially could be encoded independently in the DNA sequence.

### Regional Factors Prime Enhancers for Fast and Sustained Activation

Under ectopic NICD conditions, *m5/m8* and *sim* both produce sustained transcription profiles in the MSE and NE, whereas elsewhere they generate stochastic and “bursty” transcription. This suggests that other factors are “priming” the enhancers to respond to NICD. Good candidates are the factors involved in DV patterning at this stage, the bHLH transcription factor Twist (Twi) and/or the Rel protein Dorsal (dl) whose endogenous gradients reach the region where *m5/m8* and *sim* generate sustained profiles in response to *eve2-NICD* (Figure S6B) (Zinzen et al., 2006b). Furthermore, *m5/m8* and *sim* both contain Twist and Dorsal binding motifs (Figure S6A) and previous studies indicated that Twist is important for *sim* activity although it was not thought to regulate to *m5/m8* (Zinzen et al., 2006a).

**Figure 6:**
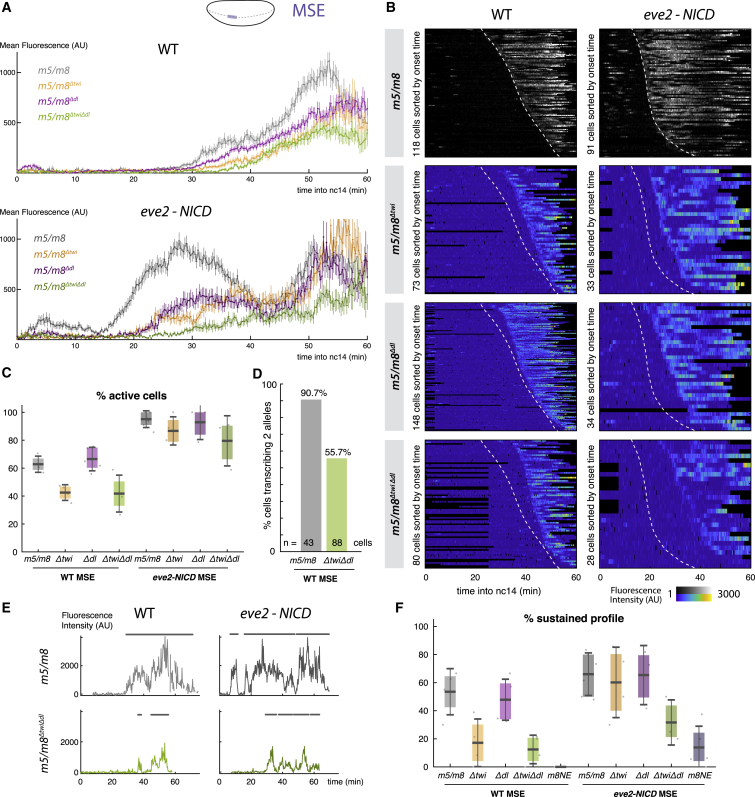
Twist and Dorsal Prime the Response of *m5/m8* to NICD (A) Mean activity levels in wild-type (top) and *eve2-NICD* (bottom) when Twist and/or Dorsal binding motifs in *m5/m8* are mutated. (B) Heatmaps of mutated enhancer activity in MSE nuclei in the wild-type and *eve2-NICD*, sorted by onset time. Dashed lines indicate onset times for unmutated enhancer. (C) Proportion of active cells in the MSE in wild-type and *eve2-NICD* embryos when Twist and/or Dorsal motifs are mutated, compared to unmutated *m5/m8*. (D) Proportion of active cells transcribing two alleles at any point, in embryos containing two copies of *m5/m8* or of *m5/m8*^Δ*twi*Δ*dl*^. (E) Examples of transcription traces from wild-type and mutated *m5/m8* in MSE nuclei from the wild-type and *eve2-NICD* embryos. Profiles from *m5/m8*^Δ*twi*Δ*dl*^ MSE cells exhibit “bursty” transcription. ON periods are marked with a gray line. (F) Proportion of MSE cells per embryo displaying a sustained profile of transcription, defined by ≥ one burst of >10 min. Median, quartiles, and SD are shown. Gray lines and heatmaps are re-plotted from Figures 3G and 3H. n = 4 (*m5/m8*^Δ*twi*^ WT), 5 (*m5/m8*^Δ*dl*^ WT), 4 (*m5/m8*^Δ*twi*Δ*dl*^ WT), 4 (*m5/m8*^Δ*twi*^*eve2-NICD*), 3 (*m5/m8*^Δ*dl*^*eve2-NICD*), 3 (*m5/m8*^Δ*twi*Δ*dl*^*eve2-NICD*), 3 (*m8NE* WT), 5 (*m8NE eve2-NICD*), and 3 (*m5/m8*^Δ*twi*Δ*dl*^ ×2) embryos. See also Figure S6.

To test if Twist and Dorsal are responsible for the different dynamics of NICD-induced transcription in the MSE versus DE (Figure 3C), we mutated the Twist and/or Dorsal binding motifs in *m5/m8* (Figure S6A). Strikingly, mutating the three Twist or two Dorsal motifs produced a delay in the start of transcription in both wild-type and *eve2-NICD* embryos. These effects were even more pronounced when Twist and Dorsal motifs were mutated together (Figure 6B). Thus, without Twist or Dorsal, *m5/m8* requires a higher threshold of NICD for activation or responds more slowly to the same threshold. The mean transcription levels were also reduced in all cases (Figure 6A).

Mutating the Twist motifs had two additional effects: the overall proportion of active cells in the MSE was reduced (Figure 6C) and few of those exhibited the sustained profile observed with wild-type *m5/m8* (Figures 6E and 6F). Instead, most displayed a “bursty” transcription profile (Figure 6E), similar to those elicited by NICD in the DE. Although the mutated Twist motifs led to bursty profiles in wild-type embryos, these effects were partially rescued when ectopic NICD was provided (Figures 6C, 6F, and S6C). When both Dorsal and Twist motifs were mutated, the proportions of active nuclei and of nuclei with sustained profiles decreased even in the presence of ectopic NICD (Figures 6C, 6F, and S6C). The decrease in the overall proportion of active cells suggests that Twist and Dorsal regulate the probability of *m5/m8* to activate transcription in response to Notch. In agreement, in embryos with two alleles of a reporter, the proportion of cells transcribing from both alleles was much lower for *m5/m8*^Δ*twi*Δ*dl*^ than for *m5/m8* (Figure 6D). Additionally, in those nuclei where both reporters were active, there was considerably more variability in the onset times for *m5/m8*^Δ*twi*Δ*dl*^ compared to *m5/m8* (Figure S6E). The results are therefore consistent with a role for Twist and Dorsal in priming the *m5/m8* enhancer to become active in response to Notch and produce sustained activity. In their absence, the ability of *m5/m8* to initiate transcription becomes much more stochastic and resembles that of *m8NE* (Figures 6F and S6D).

## Discussion

Developmental signaling pathways have widespread roles, but currently we know relatively little about how signaling information is decoded to generate the right transcriptional outcomes. We set out to investigate principles that govern how Notch activity is read by target enhancers in the living animal, using the MS2-MCP system to visualize nascent transcripts in *Drosophila* embryos and focusing on two enhancers that respond to Notch activity in the MSE. Three striking characteristics emerge. First, MSE enhancers are sensitive to changes in the levels of NICD, which modulate the transcriptional burst size rather than increasing burst frequency. Second, the activation of both MSE enhancers is highly synchronous. Indeed, within one nucleus the two enhancers become activated within few minutes of one another. Third, both MSE enhancers confer a sustained response in the wild-type context. This synchronized and persistent activity of the MSE enhancers contrasts with the stochastic and bursty profiles that are characteristics of most other enhancers that have been analyzed (Little et al., 2013, Fukaya et al., 2016, Fritzsch et al., 2018) and relies on the MSE enhancers being “primed” by regional transcription factors Twist and Dorsal. We propose that such priming mechanisms are likely to be of general importance for rendering enhancers sensitive to signals so that a rapid and robust transcriptional response is generated.

### Priming of Enhancers Sensitizes the Response to NICD

Transcription of most genes occurs in bursts interspersed with refractory periods of varying lengths that are thought to reflect the kinetic interactions of the enhancer and promoter (Bartman et al., 2016). However, the MSE enhancers appear to sustain transcription for 40–60 min, without detectable periods of inactivity, though very short off periods might not have been resolved by our assays. Calculation of the ACF in traces from these nuclei suggest very slow transcriptional dynamics (Figures S4E and S4D) (Desponds et al., 2016, Lammers et al., 2018), consistent with one long period of activity rather than overlapping short bursts. This fits with a model where promoters can exist in a permissive active state, during which many “convoys” of polymerase can be fired without reverting to a fully inactive condition (Tantale et al., 2016). The rapid successions of initiation events are thought to require Mediator complex (Tantale et al., 2016), which was also found to play a role in the NICD-mediated increase in residence time of CSL complexes (Gomez-Lamarca et al., 2018). We propose that sustained transcription from *m5/m8* and *sim* reflects a switch into a promoter permissive state, in which general transcription factors like Mediator remain associated with the promoter so long as sufficient NICD is present, allowing repeated re-initiation.

**Figure 7:**
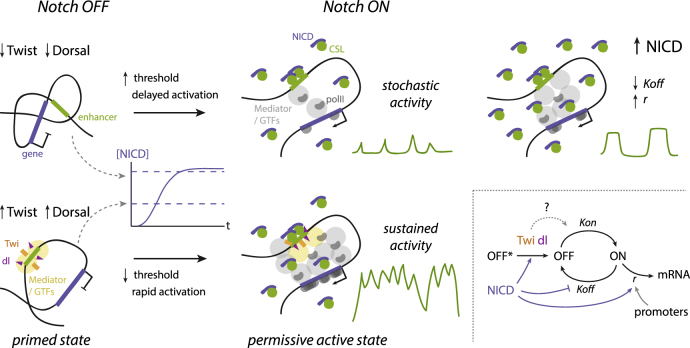
Model of Transcriptional Regulation by Notch through Enhancer Priming and Burst Size Modulation Priming by the tissue-specific factors Twist and Dorsal produces rapid activation in response to NICD and a state transition into a permissive active state in which sustained transcription can be produced without cycling between ON and OFF states. In the absence of these factors stochastic activity is produced in response to NICD. Increasing levels of NICD regulate the overall probability of the enhancer switching on (OFF^∗^ to OFF, which is also modulated by Twist and Dorsal) and increase the bursting size (higher *r* and lower *K*_*off*_). In contrast, different promoters control the initiation rate *r* but do not affect enhancer activation dynamics. The effects of Twist and Dorsal on enhancer priming might also act by modulating the same parameters of transcription.

However, the ability to drive fast and sustained activation is not a property of NICD itself. For example, when ectopic NICD was supplied, cells in many regions of the embryo responded asynchronously and underwent short bursts of activity. Furthermore, variable and less sustained cell-by-cell profiles were generated in the MSE region when the binding motifs for Twist and Dorsal in *m5/m8* were mutated. The presence of these regional factors appears to sensitize the enhancers to NICD, a process we refer to as enhancer priming. This has two consequences. First, it enables nuclei to rapidly initiate transcription in a highly coordinated manner once NICD reaches a threshold level. Second, it creates an effective “state transition” so that the presence of NICD can produce sustained activity (Figure 7). We propose a priming mechanism, rather than classic cooperativity, because Twist and Dorsal alone are insufficient to drive enhancer activity. Furthermore, since *m5/m8* and *sim* rapidly achieve sustained activity when NICD is produced, it is likely that Twist and Dorsal are required prior to NICD recruitment, although both may continue to play a role after transcription is initiated, as suggested by the lower mean levels when Twist or Dorsal motifs are mutated. Another contributory factor may be recruitment of the co-repressor complex containing CSL and Hairless, whose presence at primed enhancers could poise for activation and set the threshold (Barolo and Posakony, 2002).

Our explanation that the synchronous activation of the MSE enhancers reflects their requirements for a critical concentration of NICD is borne out by their responses when levels of NICD are increased. Notably, while *sim* and *m5/m8* had almost identical dynamics in wild-type embryos, their response to ectopic NICD differed, suggesting that they detect different thresholds. Indeed, doubling the dose of ectopic NICD further accelerated the onset time of *sim* in agreement with the model that the enhancers detect NICD levels. Threshold detection does not appear to rely on the arrangement of CSL motifs, as onset times of *m5/m8* or *sim* were unaffected by changes in the spacing of CSL paired sites. In contrast, mutating Twist- or Dorsal-binding motifs in *m5/m8* delayed the onset, arguing that these factors normally sensitize the enhancer to NICD, enabling responses at lower thresholds.

We propose that enhancer priming will be widely deployed in contexts where a rapid and consistent transcriptional response to signaling is important, as in the MSE where a stripe of cells with a specific identity is established in a short time window. In other processes where responses to Notch are more stochastic, as during lateral inhibition, individual enhancers could be preset to confer different transcription dynamics. This appears to be the case for a second enhancer from *E(spl)-C* (*m8NE*), which generates a stochastic response in the MSE cells, similar to that seen for the MSE enhancers when Twist and Dorsal sites are mutated. This illustrates that the presence or absence of other factors can toggle an enhancer between conferring a stochastic or deterministic response to signaling.

### NICD Regulates Transcription Burst Size

Manipulating NICD levels revealed that Notch-responsive enhancers act as analog devices that can measure and broadcast variations in levels. Increased NICD levels have a consistent effect on enhancer activity irrespective of the priming state of the enhancer: an increase in burst size. Transcriptional bursting has been formalized as a two-state model where the promoter toggles between ON and OFF states, conferring a transcription initiation rate when ON (Peccoud and Ycart, 1995, Larson et al., 2009). Changes in duration or frequency of bursts lead to an overall increase in transcription. Most commonly, differences in enhancer activity have been attributed to changes in switching-on probability (*Kon*), leading to changes in burst frequency (Larson et al., 2013, Senecal et al., 2014, Fukaya et al., 2016, Fritzsch et al., 2018, Lammers et al., 2018, Berrocal et al., 2018). We were therefore surprised to find that higher doses of NICD did not increase burst frequency. Instead, they produced bigger bursts, both by increasing bursting amplitude, equivalent to the rate of transcription initiation, and bursting length, indicative of the total time the enhancer stays in the ON state. Modifications to the CSL motifs also impacted on the same parameters. Thus, enhancers with paired motifs (SPS) produced larger transcription bursts than those where the motifs are further apart. This suggests that paired motifs can “use” the NICD present more efficiently, potentially because optimally configured sites increase the likelihood that at least one NICD will be bound at any time. Interestingly, even though *m5/m8* and *sim* contain different arrangements and numbers of CSL motifs they have converged to produce the same mean levels of transcription in wild-type embryos.

Two models would be compatible with the observations that effective NICD levels alter the burst size. In the first model, increasing the concentration of NICD when the enhancer is activated would create larger Pol II clusters. This is based on the observation that low-complexity activation domains in transcription factors can form local regions of high concentration of transcription factors, so-called “hubs,” which in turn are able to recruit Pol II (Mir et al., 2017, Tsai et al., 2017, Lu et al., 2018). As the lifetime of Pol II clusters appears to correlate with transcriptional output (Cho et al., 2016), the formation of larger Pol II clusters would in turn drive larger bursts. In the second model, NICD would be required to keep the enhancer in the ON state, for example, by nucleating recruitment of Mediator and/or stabilizing a loop between enhancer and promoter, which would in turn recruit Pol II in a more stochastic manner. General factors such as Mediator have been shown to coalesce into phase-separated condensates that compartmentalize the transcription apparatus (Cho et al., 2018, Sabari et al., 2018, Boija et al., 2018), and these could form in a NICD-dependent manner. Whichever the mechanism, persistence of the clusters and/or ON state requires NICD yet must be compatible with NICD having a short-lived interaction with its target enhancers (Gomez-Lamarca et al., 2018). Furthermore, the fact that the activity of *m5/m8* saturates with one *eve2-NICD* construct and cannot be enhanced by providing a more active promoter suggests that there is a limit to the size or valency of the clusters that can form.

Although unexpected, the ability to increase burst size appears to be a conserved property of NICD. Live imaging of transcription in response to the Notch homolog, GLP-1, in the *C. elegans* gonad also shows a change in burst size depending on the signaling levels (Lee et al., 2019, this issue). As the capability to modulate burst size is likely to rely on the additional factors recruited, the similarities between the effects in fly and worm argue that a common set of core players will be deployed by NICD to bring about the concentration-dependent bursting properties.

## STAR★Methods

### Key Resources Table

**Table:** 

REAGENT or RESOURCE	SOURCE	IDENTIFIER
**Experimental Models: Organisms/Strains**		

*D. melanogaster* His2Av-mRFP; nos-MCP-eGFP	Bloomington Drosophila Stock Center	RRID:BDSC_60340
*D. melanogaster* nos-MCP-eGFP (II)	Bloomington Drosophila Stock Center	RRID:BDSC_63821
*D. melanogaster* His2Av-RFP (III)	Bloomington Drosophila Stock Center	RRID:BDSC_23650
*D. melanogaster* Df(3R)Dl^FX3^	Vässin and Campos-Ortega, 1987	N/A
*D. melanogaster* Neur[11]	Bloomington Drosophila Stock Center	RRID:BDSC_2747
*D. melanogaster* TTG (TM3, twi-Gal4, UAS-2xeGFP, Sb, Ser)	Bloomington Drosophila Stock Center	RRID:BDSC_6663
*D. melanogaster* Ovo-FLP	Bloomington Drosophila Stock Center	RRID:BDSC_8727
*D. melanogaster* betaTub85D-FLP	Bloomington Drosophila Stock Center	RRID:BDSC_7196
*D. melanogaster* Vas-int; attP40	Bischof et al., 2007	N/A
*D. melanogaster* vas-int; attp-51D	Bloomington Drosophila Stock Center	RRID:BDSC_24483 (RFP cre-ed out)
*D. melanogaster* vas-int; attp86Fb	Bloomington Drosophila Stock Center	RRID:BDSC_24749
*D. melanogaster* m5/m8-peve-MS2-lacZ (II)	This study	N/A
*D. melanogaster* m5/m8-hsp70-MS2-lacZ (II)	This study	N/A
*D. melanogaster* m5/m8-pm5-MS2-lacZ (II)	This study	N/A
*D. melanogaster* m5/m8-pm6-MS2-lacZ (II)	This study	N/A
*D. melanogaster* m5/m8-pm7-MS2-lacZ (II)	This study	N/A
*D. melanogaster* m5/m8-pm8-MS2-lacZ (II)	This study	N/A
*D. melanogaster* m5/m8-psimE-MS2-lacZ (II)	This study	N/A
*D. melanogaster* sim-peve-MS2-lacZ (II)	This study	N/A
*D. melanogaster* sim-psimE-MS2-lacZ (II)	This study	N/A
*D. melanogaster* m8NE-peve-MS2-lacZ (II)	This study	N/A
*D. melanogaster* sim^SPS^-peve-MS2-lacZ (II)	This study	N/A
*D. melanogaster* m5/m8^insSPS^-peve-MS2-lacZ (II)	This study	N/A
*D. melanogaster* m5/m8^Δtwi^-peve-MS2-lacZ (II)	This study	N/A
*D. melanogaster* m5/m8^Δdl^-peve-MS2-lacZ (II)	This study	N/A
*D. melanogaster* m5/m8^ΔtwiΔdl^-peve-MS2-lacZ (II)	This study	N/A
*D. melanogaster* m5/m8-peve-MS2-lacZ (III)	This study	N/A
*D. melanogaster* eve2-NICD (II, 25C)	This study	N/A
*D. melanogaster* eve2-NICD (II, 51D)	This study	N/A

**Oligonucleotides**		

Oligonucleotides for cloning of enhancers and promoters in MS2 reporters and mutagenesis	This study	Table S1

**Recombinant DNA**		

pLacZ2-attB	Bischof et al., 2013	N/A
pCR4-24XMS2SL-stable	Addgene	RRID:Addgene_31865
22FPE (eve.st.2-Pme)	Kosman, and Small, 1997	N/A
pGEM-t-easy	Promega	N/A
pattB	Bischof et al., 2013	N/A
pMT-NICD	Bray lab	N/A

**Software and Algorithms**		

Tracking code	This study	https://github.com/juliafs93/FryEmbryo3DTracking
Modelling changes in transcription	This study	https://github.com/juliafs93/FFR_ACF
Matlab 2018a	Mathworks	N/A

### Lead Contact and Materials Availability

Further information and requests for resources and reagents should be directed to and will be fulfilled by the Lead Contact, Sarah J. Bray (sjb32@cam.ac.uk).

### Experimental Model and Subject Details

#### Experimental Animals

*Drosophila melanogaster* flies were grown and maintained on food consisting of the following ingredients:

Glucose 76g/l, Cornmeal flour 69g/l, Yeast 15g/l, Agar 4.5g/l, Methylparaben 2.5ml/l. Embryos were collected on apple juice agar plates with yeast paste. Animals of both sexes were used for this study.

#### Cloning and Transgenesis

##### Generation of MS2 Reporter Constructs

*MS2* loops were inserted upstream of a *lacZ* transcript within the 5’UTR and then the resulting reporter was combined with different enhancers and promoters. 24 *MS2* loops were cloned from *pCR4-24XMS2SL-stable* (Addgene #31865) into *pLacZ2-attB* (Bischof et al., 2013) using *Eco* RI sites. The *m5/m8*, *sim* and *m8NE* enhancers (Zinzen et al., 2006a, Kramatschek and Campos-Ortega, 1994) were amplified from genomic DNA and cloned into *pattB-MS2-LacZ* using *Hin*dIII/*Age*I sites (primers in Table S1). Subsequently the promoters *hsp70*, *peve*, *pm5*, *pm6*, *pm7*, *pm8* and *psimE* were cloned by Gibson Assembly (Gibson, 2011) in *pattB-m5/m8-MS2-LacZ*, *pattB-sim-MS2-LacZ* and/or *pattB-m8NE-MS2-LacZ* (primers in Table S1) using the *Age*I restriction site and incorporating a *Eag*I site.

Su(H), Twi, dl and Sna binding motifs were identified using ClusterDraw2 using the PWM from the Jaspar database for each transcription factor. Motifs with scores higher than 6 and p values < 0.001 were selected. Primers to create *sim*^*SPS*^, *m5/m8*^*insSPS*^, *m5/m8*^Δ*twi*^, *m5/m8*^Δ*dl*^ and *m5/m8*^Δ*twi*^ ^Δ*dl*^ are detailed in Table S1. All mutations were first introduced by Gibson Assembly in the enhancers contained in *pCR4* plasmids and then transferred to *pattB-peve-MS2-lacZ* using *Hin*dIII and *Age*I sites.

The following constructs have been generated and inserted by ΦC31 mediated integration (Bischof et al., 2007) into an *attP* landing site in the second chromosome – *attP40*, 25C – to avoid positional effects in the comparisons: *pattB-m5/m8-peve-MS2-LacZ*, *pattB-m5/m8-hsp70-MS2-LacZ*, *pattB-m5/m8-pm5-MS2-LacZ*, *pattB-m5/m8-pm6-MS2-LacZ*, *pattB-m5/m8-pm7-MS2-LacZ*, *pattB-m5/m8-pm8-MS2-LacZ*, *pattB-m5/m8-psimE- MS2-LacZ*, *pattB-m8NE-peve-MS2-LacZ*, *pattB-sim-peve-MS2-LacZ*, *pattB-sim-psimE-MS2-LacZ*, *pattB-sim*^*SPS*^*-peve-MS2-LacZ*, *pattB-m5/m8*^*insSPS*^*-peve-MS2-LacZ*, *pattB-m5/m8*^*Δtwi*^*-peve-MS2-LacZ*, *pattB-m5/m8*^*Δdl*^*-peve-MS2-LacZ* and *pattB-m5/m8*^*ΔtwiΔdl*^*-peve-MS2-LacZ*. *pattB-m5/m8-peve-MS2-LacZ* was also inserted in a different landing site in the third chromosome - *attP86Fb* (BDSC # 24749).

##### Expression of Ectopic NICD

To generate *eve2-NICD* the plasmid 22FPE (Kosman and Small, 1997), which contains 2 copies of the *eve2* enhancer with five high affinity *bicoid* sites, FRT sites flanking a transcription termination sequence and the *eve 3’UTR*, was transferred to *pGEM-t-easy* using *Eco*RI sites and from there to *pattB* (Bischof et al., 2013) using a *Not*I site. The NICD fragment from Notch was excised from an existing *pMT-NICD* plasmid and inserted in *pattB-22FPE* through the *Pme*I site to create the *pattB-eve2x2-peve-FRT-STOP-FRT-NICD-eve3’UTR* construct (referred to as *eve2-NICD*). This was inserted into the *attP* landing site at 51D in the second chromosome. To increase the amount of ectopic NICD produced, the same *eve2-NICD* construct was also inserted in the *attP40* landing site at 25C and recombined with *eve2-NICD* 51D to produce 2x*eve2-NICD*. Sequences of all generated plasmids are available in a benchling repository (https://benchling.com/juliafs/f_/AyerQ4a4-dynamics-notch-transcription-paper/).

#### Fly Strains and Genetics

To observe the expression pattern and dynamics from *m5/m8-peve*, *sim-peve*, *m8NE-peve* and the different promoter combinations (Figures 1 and S1) females expressing His2av-RFP and MCP-GFP (BDSC #60340) in the maternal germline were crossed with males expressing the *MS2-lacZ* reporter constructs.

To test expression from *m5/m8-peve* in the *Dl* and *neur* mutant backgrounds, *His2Av-RFP* from *His2av-RFP; nos-MCP-GFP* (BDSC #60340) was recombined with *nos-MCP-GFP* in the second chromosome (BDSC #63821) and combined with a deficiency encompassing the *Dl* gene (*Df(3R)Dl*^*FX3*^, (Vässin and Campos-Ortega, 1987)) or a *neuralized* loss of function allele (*neur*^*[11]*^, BDSC #2747). *m5/m8-peve-MS2-lacZ* was also combined with the *Dl* and *neur* alleles and mutant embryos were obtained from the cross *His2Av-RFP,nos-MCP-GFP ; mut / TTG* x *m5/m8-peve-MS2-lacZ ; mut / TTG*. Homozygous mutant embryos for *Dl* or *neur* were selected by the lack of expression from the *TTG* balancer (*TM3-twi-GFP*, BDSC #6663).

To observe transcription from two MS2 reporters in each cell (Figures 2 and S2) *His2Av-RFP* (BDSC #23650) was recombined with *nos-MCP-GFP* (from BDSC #60340) in the third chromosome and combined with *m5/m8- peve*, *sim-peve* or *m5/m8*^Δ*twi*^ ^Δ*dl*^ *-peve* MS2 reporters. *m5/m8-peve* x2, *sim-peve* x2 and *m5/m8*^Δ*twi*^ ^Δ*dl*^ *-peve* x2 embryos were obtained from the stocks *m5/m8-peve-MS2-LacZ ; His2Av-RFP,nos-MCP-GFP*, *sim-peve-MS2-LacZ* ; *His2Av-RFP,nos-MCP-GFP* and *m5/m8*^Δ*twi*^ ^Δ*dl*^ *-peve-MS2-LacZ ; His2Av-RFP,nos-MCP-GFP*, respectively; while *m5/m8-peve* + *sim-peve* embryos were obtained from crossing *sim-peve-MS2-LacZ ; His2Av-RFP,nos-MCP-GFP* females with *m5/m8-peve-MS2-LacZ* males.

To observe transcription from MS2 reporters in conditions of ectopic Notch activity the *FRT-STOP-FRT* cassette had to be first removed from the *eve2-NICD* construct by expression of a flippase in the germline. To do so flies containing *ovo-FLP* (BDSC #8727), *His2Av-RFP* and *nos-MCP-GFP* were crossed with others containing *eve2-FRT-STOP-FRT-NICD*, *His2Av-RFP* and *nos-MCP-GFP*. The female offspring of this cross (*ovo-FLP/+ ; eve2- FRT-STOP-FRT-NICD/+ ; His2Av-RFP, nos-MCP-GFP*) induced FRT removal in the germline and were crossed with the MS2 reporters to obtain embryos expressing ectopic NICD. We note that only half of the embryos present the *eve2-NICD* chromosome, which could be distinguished by ectopic MS2 activity and an ectopic cell division of all the cells in the *eve2* stripe after gastrulation. The other 50% embryos obtained from this cross were used as the wild type controls. This strategy was used to observe transcription from *m5/m8-peve*, *sim-peve*, *m8NE-peve*, *m5/m8-pm5*, *sim*^*SPS*^ *-peve*, *m5/m8*^*insSPS*^ *-peve*, *m5/m8*^Δ*twi*^ *-peve*, *m5/m8*^Δ*dl*^ *-peve* and *m5/m8*^Δ*twi*^ ^Δ*dl*^ *-peve*. To measure transcription from 2x*eve2-NICD* (Figures 4 and S4) removal of the *FRT-STOP-FRT* cassette was induced from the male germline to avoid recombination. To do so, *betaTub85D-FLP* (BDSC #7196) females were crossed with 2x*eve2-NICD* males and the male offspring of this cross (*betaTub85D-FLP/Y* ; *2xeve2-NICD* /+), which induces FRT removal in the germline, were crossed with *m5/m8-peve-MS2-lacZ ; His2AvRFP*, *nos-MCP-GFP* or *sim-peve-MS2-lacZ ; His2AvRFP, nos-MCP-GFP* females. As in the previous strategy, only half of the embryos presented the 2x*eve2-NICD* chromosome and were distinguished by the ectopic activity. To express two *m5/m8-peve* reporters in conditions of ectopic NICD activity, *m5/m8-peve* and *eve2-NICD* were recombined in the second chromosome and embryos were obtained by crossing *m5/m8-peve-MS2-lacZ ; His2AvRFP, nos-MCP-GFP* females with *betaTub85D-FLP/Y ; m5/m8-peve*, *eve2-NICD* /+ males. Embryos were selected by the presence of two MS2 spots in each cell, which also ectopically expressed NICD.

### Method Details

#### Live Imaging

Embryos were dechorionated in bleach and mounted in Voltalef medium (Samaro) between a semi-permeable membrane and a coverslip. The ventral side of the embryo was facing the coverslip in all videos except when looking at transcription in the DE region, for which they were mounted laterally. Videos were acquired in a Leica SP8 confocal using a 40x apochromatic 1.3 objective and the same settings for MCP-GFP detection: 40mW 488nm argon laser detected with a PMT detector, pinhole airy=4. Other settings were slightly different depending on the experiment. To observe transcription in the whole embryo (Figures 1 and S1) settings were: 3% 561nm laser, 0.75x zoom, 800x400 pixels resolution (0.48μm/pixel), 19 1μm stacks, final temporal resolution of 10 seconds/frame). To observe transcription from 2 MS2 alleles simultaneously (Figures 2, S2, and S6E) settings were: 2% 561nm laser, 1.5x zoom, 800x400 pixels resolution (0.24μm /pixel), 29 1μm stacks, final temporal resolution of 15s/frame). In all other experiments with ectopic NICD a ∼150x150μm window anterior to the center of the embryo was captured. Settings were: 2% 561nm laser, 2x zoom, 400x400 pixels resolution (0.36μm /pixel), 29 1μm stacks, final temporal resolution of 15s/frame). All images were collected at 400Hz scanning speed in 12 bits.

### Quantification and Statistical Analysis

#### Image Analysis

Videos were analyzed using custom Matlab (Matlab R2018a, Mathworks) scripts (available at https://github.com/juliafs93/FryEmbryo3DTracking)

Briefly, the His2Av-RFP signal was used to segment and track the nuclei in 3D. Each 3D stack was first filtered using a median filter, increasing the contrast based on the profile of each frame to account for bleaching and a fourier transform log filter (Garcia et al., 2013). Segmentation was performed by applying a fixed intensity threshold, 3D watershed accounting for anisotropic voxel sizes (Mishchenko, 2015) to split merged nuclei and thickening each segmented object. Nuclei were then tracked by finding the nearest object in the previous 2 frames which was closer than 6 μm. If no object was found, that nuclei was kept with a new label, and only one new object was allowed to be tracked to an existing one. After tracking, the 3D shape of each nucleus in each frame was used to measure the maximum fluorescence value in the GFP channel, which was used as a proxy of the spot fluorescence. We note than when a spot cannot be detected by eye this method detects only background, but the signal:background ratio is high enough that the subsequent analysis allows to classify confidently when the maximum value is really representing a spot.

In experiments with two MS2 reporters the maximum intensity pixel per nucleus does not allow to separate transcription from the two alleles. To do so, the 3D Gaussian spot detection method from (Garcia et al., 2013) was implemented in the existing tracking, such that each spot was segmented independently and associated with the overlapping nuclei. In this manner only active transcription periods were detected and no further processing of the traces was required.

#### MS2 Data Processing

From the previous step we obtained the fluorescent trace of each nuclei over time. Only nuclei tracked for more than 10 frames were retained. First nuclei were classified as inactive or active. To do so the average of all nuclei (active and inactive) was calculated over time and fitted to a straight line. A median filter of 3 was applied to each nuclei over time to smooth the trace and ON periods were considered when fluorescent values were 1.2 times the baseline at each time point. This produced an initial segregation of active (nuclei ON for at least 5 frames) and inactive nuclei. These parameters were determined empirically on the basis that the filters retained nuclei with spots close to background levels and excluded false positives from bright background pixels. The mean fluorescence from MCP-GFP in the inactive nuclei was then used to define the background baseline and active nuclei were segregated again in the same manner. The final fluorescence values in the active nuclei were calculated by removing the fitted baseline from the maximum intensity value for each, and normalizing for the percentage that the MCP-GFP fluorescence in inactive nuclei decreased over time to account for the loss of fluorescence due to bleaching. Nuclei active in cycles before nc14 were discarded based on the timing of their activation.

In all videos, time into nc14 was considered from the end of the 13th syncytial division. When this was not captured the videos were synchronized by the gastrulation time. Plots showing mean fluorescent levels were obtained by calculating the mean and SEM of all fluorescent traces for multiple embryos aligned by the beginning of nc14. Calculating the mean levels of multiple embryos taken individually returned very similar profiles, indicating there is little embryo-to-embryo variability. In Figures 1C and 3C the total mRNA production per cell (in AU) was calculated by adding all the normalized fluorescent intensities for each nucleus.

Each embryo was classified into the 4 regions (ME, MSE, NE and DE) by drawing rectangular shapes in a single frame and finding which centroids overlapped with each region. In *eve2-NICD* these regions along the DV axis were defined within the *eve2* stripe (∼ 6-7 cells wide in all videos). In wild type embryos ME and MSE regions were drawn in the whole field of view (∼ 150x150 μm anterior half of the embryo).

#### Definition of Bursting Properties

Bursts were defined as periods were the median-filtered signal was higher than 1.2 times the baseline for at least 5 frames within a period from 15 min into nc14. These defined the burst duration and the time off between bursts. The amplitude was defined as the mean value within each burst period. The proportion of active cells was defined as the percentage of cells that switch on at any point after 15 min in each of the defined regions. ’Sustained’ transcription was defined as nuclei with at least one burst longer than 10 min. This was based on analyzing regions where separated burst of activity were detected (mesoderm and dorsal ectoderm) where most bursts were <10 min. Off periods shorter than circa 2 mins would not have been resolved because the MS2 loops were positioned within the 5’UTR and the limit of resolution depends on the time taken for a Pol II molecule to complete transcription.

Onsets and ends of transcription were defined as the beginning of the first burst and the end of the last respectively (also starting at 15 min into nc14). In Figures 2 and S2 to be more precise in measuring the onsets and end points of transcription for both MS2 alleles they were scored manually as the first and last frame a spot is detected and randomly assigned ’allele 1’ or ’allele 2’. The total variability was the variance of all onsets or end points, combining both alleles. The extrinsic variability was calculated as the covariance of onsets and ends between alleles 1 and 2. The remaining (total - covariance) corresponds to the intrinsic variability within each cell.

#### Statistical Analysis

In figure legends, n number indicates number of embryos imaged for each biological condition. Where appropriate, n number next to heatmaps indicates total number of cells combining all embryos for each biological condition. Plots showing mean levels of transcription and SEM (standard error of the mean) combine all traces from multiple embryos from the same biological condition. Violin plots show the bursting properties (amplitude, burst duration and off period) for each independent burst in all traces in multiple embryos, therefore the n number can be significantly greater than the number of cells in each condition. Because these properties do not follow a normal distribution, their statistical significance was tested with two Kolmogorov-Smirnov test. Levels of significance are indicated in the figure legends.

#### Modelling Changes in Kinetic Parameters of Transcription

We used a two-state promoter model of transcriptional activation in which the promoter switches between OFF and ON with constants *K*_*on*_ and *K*_*off*_ and releases mRNAs at a rate *r* when the promoter is ON (Figure 4E). This model also accounts for the residence time of polymerase on DNA while transcribing the gene (the elongation time *T*), so it is capturing what the MS2 system detects, ie. the number of nascent mRNA on the gene, rather than overall levels of mRNA in the cell. We take as a starting point expressions from (Choubey et al., 2015) for the mean and variance of the number of nascent mRNAs 〈*m*〉 in steady state:

(1) $$\begin{array}{ccc} \langle m\rangle & = & \frac{rTK_{on}}{K_{on}+K_{off}} \end{array}$$

(2) $$\begin{array}{ccc} Var\left(m\right) & = & \langle m\rangle \left[1+\frac{2rK_{off}}{{\left(K_{on}+K_{off}\right)}^{2}}+\frac{2rK_{off}}{{\left(K_{on}+K_{off}\right)}^{3}}\left(\frac{e^{-T\left(K_{on}+K_{off}\right)}-1}{T}\right)\right] \end{array}$$

We take the elongation time, *T*, to be fixed for a given gene. Thus, according to Equation 1, the levels of transcription could increase in three ways: by increasing *r*, increasing *K*_*on*_, or decreasing *K*_*off*_.

Thus, because of this degeneracy, observing a change in 〈*m*〉 is alone insufficient to determine which underlying bursting parameter is being tuned to drive that change. However, we can make progress by incorporating the intrinsic noise of transcription into our analysis, since Equation 2 indicates that changes to bursting parameters that have equivalent effects on the mean may nonetheless lead to different noise signatures. To do this, we calculate the Fano factor, which is defined as the variance divided by the mean:

(3) $$\begin{array}{ccc} Fano\left(m\right) & = & \frac{Var\left(m\right)}{\langle m\rangle } \end{array}$$

(4) $$\begin{array}{ccc} \;\;\; & = & 1+\frac{2rK_{off}}{{\left(K_{on}+K_{off}\right)}^{2}}+\frac{2rK_{off}}{{\left(K_{on}+K_{off}\right)}^{3}}\left(\frac{e^{-T\left(K_{on}+K_{off}\right)}-1}{T}\right) \end{array}$$

Where we see that the expression for the Fano factor is identical to the quantity inside the brackets in Equation 2.

Next, we examine how changes to each bursting parameter in turn will affect the Fano factor and Mean, respectively, demonstrating how these signatures can be used to uncover the drivers of observed changes between different experimental conditions.

**Pol II Initiation Rate (***r***)**

We start by considering the case when *r* is modulated. In the discussions that follow, we assume a situation in which we are comparing two experimental conditions that exhibit observable differences in their mean rate of expression, 〈*m*〉:

(5) *α*〈*m*_1_〉 = 〈*m*_2_〉

Our goal is to determine whether the modulation of specific parameters corresponds reliably with changes in the mean and Fano factor. To do this, we undertake analysis of the functional form of the partial derivatives of these empirical measures with respect to each parameter.

From Equation 1, we have:

(6) $$\frac{\partial \langle m\rangle }{\partial r}=\frac{TK_{on}}{\kappa }$$

(7) $$\frac{\partial \langle m\rangle }{\partial r}\;>\;0$$

Where, for convenience, we have introduced the shorthand *κ* = *K*_*on*_ + *K*_*off*_. So we see that 〈*m*〉 is monotonic with

*r*: an increase in *r* always leads to an increase in the mean (and vice versa). The strict inequality applies because the right-hand-side of Equation 6 can be zeros *if* no expression occurs. For the Fano factor, we have:

(8) $$\frac{\partial Fano}{\partial r}=\frac{2K_{off}}{\kappa^{2}}\left(1+\frac{e^{-\kappa T}-1}{\kappa T}\right)$$

(9) $$\frac{\partial Fano}{\partial r}\;\geq \;0$$

Unlike the mean, it is possible that a change in *r* could lead to *no* observable modulation in the Fano factor; however, this only holds for exceptionally small values of *κT*. More importantly, we see that it is impossible for the Fano factor to decrease when *r* is increased. Thus, we conclude that an increase in *r* must coincide with an increase in both the mean rate of expression and in the Fano factor, ie. the ratio between the Fano factors *Fano*(*m*_2_) and *Fano*(*m*_1_) where 〈*m*_2_〉= *α*〈*m*_1_〉 would always be greater than 1 (Figure S3D, top panel).

**Activation Rate (***Kon)*

As with *r*, we begin by examining how 〈*m*〉 changes in response to a change in *Kon* :

(10) $$\begin{array}{ccc} \frac{\partial \langle m\rangle }{\partial K_{on}} & = & \frac{rT}{\kappa }-\frac{rTK_{on}}{\kappa^{2}} \end{array}$$

(11) $$\begin{array}{ccc} \;\; & = & \frac{rT}{\kappa }\left(1-\frac{K_{on}}{\kappa }\right) \end{array}$$

(12) $$\begin{array}{ccc} \frac{\partial \langle m\rangle }{\partial K_{on}} & \geq & 0 \end{array}$$

Thus, as with *r*, the mean rate of expression increases monotonically in response to increases in *K_on_*. Next, for the Fano factor, we have:

(13) $$\begin{array}{ccc} \frac{\partial Fano}{\partial k_{on}} & = & 2rK_{off}\left(-\kappa^{-3}\left(2+e^{-\kappa T}\right)+\frac{3\kappa^{-4}}{T}\left(1-e^{-\kappa T}\right)\right) \end{array}$$

(14) $$\begin{array}{ccc} \;\;\; & = & -\frac{2rK_{off}}{\kappa^{3}}\left(2+e^{-\kappa T}-\frac{3\left(1-e^{-\kappa T}\right)}{\kappa T}\right) \end{array}$$

To gain further insight, we need to examine limiting cases for the quantity *κT*, which encodes the relative magnitude of the elongation time and switching rates, and which dictates the noise characteristics of the system.

We start with the case where *κT ≪* 1:

(15) $$\begin{array}{ccc} \frac{\partial Fano}{\partial k_{on}} & \approx & -\frac{2rK_{off}}{\kappa^{3}}\left(2+1-\kappa T-\frac{3\left(1+\kappa T-1\right)}{\kappa T}\right) \end{array}$$

(16) $$\begin{array}{ccc} \;\;\; & \approx & -\frac{2rK_{off}}{\kappa^{3}}\left(3-\kappa T-3\right) \end{array}$$

(17) $$\begin{array}{ccc} \;\;\; & \approx & -\frac{2rK_{off}}{\kappa^{3}}\left(0\right) \end{array}$$

(18) $$\begin{array}{ccc} \;\;\; & \approx & -\frac{2rK_{off}}{\kappa^{3}}\left(3-\kappa T-3\right) \end{array}$$

(19) $$\begin{array}{ccc} \frac{\partial Fano}{\partial k_{on}} & \approx & 0 \end{array}$$

For the opposite limit, where *κT ≫* 1, we have:

(20) $$\begin{array}{ccc} \frac{\partial Fano}{\partial k_{on}} & \approx & -\frac{2rK_{off}}{\kappa^{3}}\left(2+0-\frac{3\left(1-0\right)}{\kappa T}\right) \end{array}$$

(21) $$\begin{array}{ccc} \;\;\; & \approx & -\frac{4rK_{off}}{\kappa^{3}} \end{array}$$

(22) $$\begin{array}{ccc} \frac{\partial Fano}{\partial k_{on}} & \leq & 0 \end{array}$$

So we see that, an increase in 〈m〉 that is driven by an increase in *K_on_* will coincide with a decrease in the Fano factor. Thus, unlike *r*, where the signs of the change in the mean and Fano factor are the same, we find that the signs of the changes in the mean and Fano factor are opposite in the case of changes driven by *K*_*on*_, ie. the ratio between the Fano factors Fano(m2) and Fano(m1) where 〈m2〉 = α〈m1〉 would always be smaller than 1 (Figure S3D, middle panel).

**Off Rate (***K*_*off*_)

For the mean, we have:

(23) $$\begin{array}{ccc} \frac{\partial \langle m\rangle }{\partial K_{off}} & = & -\frac{rK_{on}}{\kappa^{2}} \end{array}$$

(24) $$\begin{array}{ccc} \; & \;\; & \frac{\partial \langle m\rangle }{\partial K_{off}}\;\leq \;0 \end{array}$$

Thus, as expected, an increase in *K*_*off*_ leads to a decrease in 〈m〉. In keeping with our treatment in the case of

*K*_*on*__,_ we next examine the functional form of the Fano factor in the small and large *κT* limits. For *κT* ≪ 1, we expand about *κT* = 0 to obtain an expression for the Fano factor:

(25) $$\begin{array}{ccc} Fano & \approx & 1+\frac{2rK_{off}}{{\left(K_{on}+K_{off}\right)}^{2}}+\frac{2rK_{off}}{{\left(K_{on}+K_{off}\right)}^{3}}\left(\frac{1-\kappa T-1}{T}\right) \end{array}$$

(26) $$\begin{array}{ccc} \;\; & \approx & 1 \end{array}$$

Thus, consistent with our findings for *K*_*on*_ the Fano factor is largely insensitive to changes in *K*_*off*_ for small *κT*. This holds for *r* as well, though we did not state so explicitly above. Next, we approximate the large *κT* limit by setting *e*^*−kT*^ = 0:

(27) $$\begin{array}{ccc} Fano & \approx & 1+\frac{2rK_{off}}{\kappa^{2}}+\frac{2rK_{off}}{\kappa^{3}}\left(\frac{0-1}{T}\right) \end{array}$$

(28) $$\begin{array}{ccc} \;\; & \approx & 1+2r\left(\frac{K_{off}}{\kappa^{2}}-\frac{K_{off}}{\kappa^{2}}\frac{1}{\kappa T}\right) \end{array}$$

(29) $$\begin{array}{ccc} \;\; & \approx & 1+2r\left(\frac{K_{off}}{\kappa^{2}}\right) \end{array}$$

Differentiating, we obtain:

(30) $$\begin{array}{ccc} \frac{\partial Fano}{\partial_{off}} & \approx & 2r\left(\frac{1}{\kappa^{2}}-\frac{2K_{off}}{\kappa^{3}}\right) \end{array}$$

(31) $$\begin{array}{ccc} \;\; & \approx & \frac{2r}{\kappa^{2}}\left(1-\frac{2K_{off}}{\kappa }\right) \end{array}$$

The expression above reveals that, unlike *r* and *K_on_,* the direction of the change of the Fano factor in response to a change in *K*_*off*_ not fixed, but depends upon the relative sizes of *K*_*on*_ and *K*_*off*_, ie. the ratio between the Fano factors *Fano*(*m*_2_) and *Fano*(*m*_1_) where 〈*m*_2_〉 = *α*〈*m*_1_〉 could be smaller or greater than 1 (Figure S3D, bottom panel). Numerical simulations confirm this result.

**Stochastic simulations**

We next tested with simulations whether the Fano factor ratio can be used as a diagnostic tool of the underlying changes in the mean. We used stochastic simulations of transcription based on the Gillespie algorithm (Gillespie 1976) of the same two-state promoter model but using additional parameters to more resemble the biological MS2 data (accounting for the time MS2 loops are detected, acquisition time and adding experimental noise, Figure S3F).

We then tested whether we could recover the same trends in Fano factor ratios in the simulation as expected from the mathematical model. Indeed, using a variety of starting parameters we could recover similar Fano factor values as expected from the mathematical model (Figure S3D). However, given that changes in *K*_*off*_ can produce Fano factor ratios greater or smaller than 1, calculation of the Fano factor and comparing whether it is greater or smaller than 1 alone is not sufficient to infer which parameter is being modified to produce the observed changes in the mean.

**Utilizing the Autocorrelation Function (ACF)**

The results of our analysis thus far indicate that modulations in *r* and *K_on_* lead to distinct, well defined signatures in mean and Fano factors of experimentally observed expression levels. However, the degeneracy of the Fano factor shift with respect to changes in *K*_*off*_ necessitates the incorporation of an additional observable, if we are to be able to distinguish the underlying drivers of changes between experimental conditions. To this end, we utilize the empirical Autocorrelation Function of our experimental MS2 traces.

The ACF function provides information about the speed of the system and the elongation rate (Desponds et al., 2016, Lammers et al., 2018). Intuitively, the more rapid the time scale with which the system switches between activity states (the larger *κ* is), the faster the ACF decays. We used the same simulations to test if the autocorrelation function changes in different ways depending on the modified parameters, to help distinguishing between the 3 scenarios to increase the mean. If the dynamics are fast (Figure S3E, right column, *K*_*on1*_ = 0.1 s^*−*1^ and *K*_*on1*_ = 0.2 s^*−*1^) no changes in the ACF were observed in any of the three cases. When the dynamics are slower (Figure S3E, left column, *K*_*on1*_ = 0.01 s^*−*1^ and *K*_*on1*_ = 0.02 s^*−*1^), then the AC function shifts to the right (from 〈*m*_1_〉 to 〈*m*_2_〉) when *K*_*off*_ decreases. No changes are observed when *r* or *K*_*on*_ increase.

Therefore looking at both the Fano factor ratio and the autocorrelation function (when the dynamics are slow enough), provides enough information to distinguish between the three ways in which the mean can change (Figure S3B):

- increase in *r*: FFRatio *>* 1 and no change in ACF
- increase in *K_on_* : FFRatio *<* 1 and no change in ACF
- decrease in *K*_*off*_ : FFRatio *<* 1 or > 1 and shift to the right in ACF

**Estimating Fano factor from empirical data**

When applied to real MS2 traces, raw fluorescence profiles from each cell were processed by applying a median filter of 3, removing the background baseline and normalizing for bleaching as described in the MS2 data processing section. When the onset of transcription was different between experiments (eg. WT vs *eve2-NICD*) they were shifted to compare equivalent times. The Fano factor was calculated as the intrinsic variability divided by the mean over time:

(32) $$\begin{array}{ccc} Fano & = & \frac{\sigma_{i}^{2}}{\langle m\rangle } \end{array}$$

(33) $$\begin{array}{ccc} \;\; & = & \frac{Var\left(m\right)-CoVar\left(m\right)}{\langle m\rangle } \end{array}$$

The intrinsic component was calculated by subtracting an estimation of the extrinsic variability form the total noise. The contribution from the extrinsic noise, normally calculated from the covariance of two transcription traces from the same cell, was calculated by using neighbouring nuclei as proxi of two loci in the same cell and calculating their covariance. Using the experiments where two MS2 reporters are present in each cell we validated the contribution from extrinsic noise is equivalent within cell and across neighbouring cells. Both FFRatio and ACF were calculated by doing 50 bootstraps of all available traces and calculating the mean and SD.

### Data and Code Availability

Scripts for tracking and analysis of MS2 videos are available at https://github.com/juliafs93/FryEmbryo3DTracking.

The code developed for the modelling approach to infer changes in parameters of transcription causing changes in mean levels of transcription is available at https://github.com/juliafs93/FFR_ACF.

## Supplemental Information

Document S1. Figures S1–S6 and Table S1

Document S2. Article plus Supplemental Information
