# Supplementary material for: Enhancer Priming Enables Fast and Sustained Transcriptional Responses to Notch Signaling
Source: Dev Cell. 2019 Aug 19;50(4):411–425.e8. doi: 10.1016/j.devcel.2019.07.002 (PMC6706658; doi:10.1016/j.devcel.2019.07.002)
Supplement: Document S1. Figures S1–S6 and Table S1 [file mmc1.pdf]

Developmental Cell, Volume *50*

Supplemental Information

Enhancer Priming Enables Fast and Sustained Transcriptional Responses to Notch Signaling

Julia Falo-Sanjuan, Nicholas C. Lammers, Hernan G. Garcia, and Sarah J. Bray

Supplemental Figures

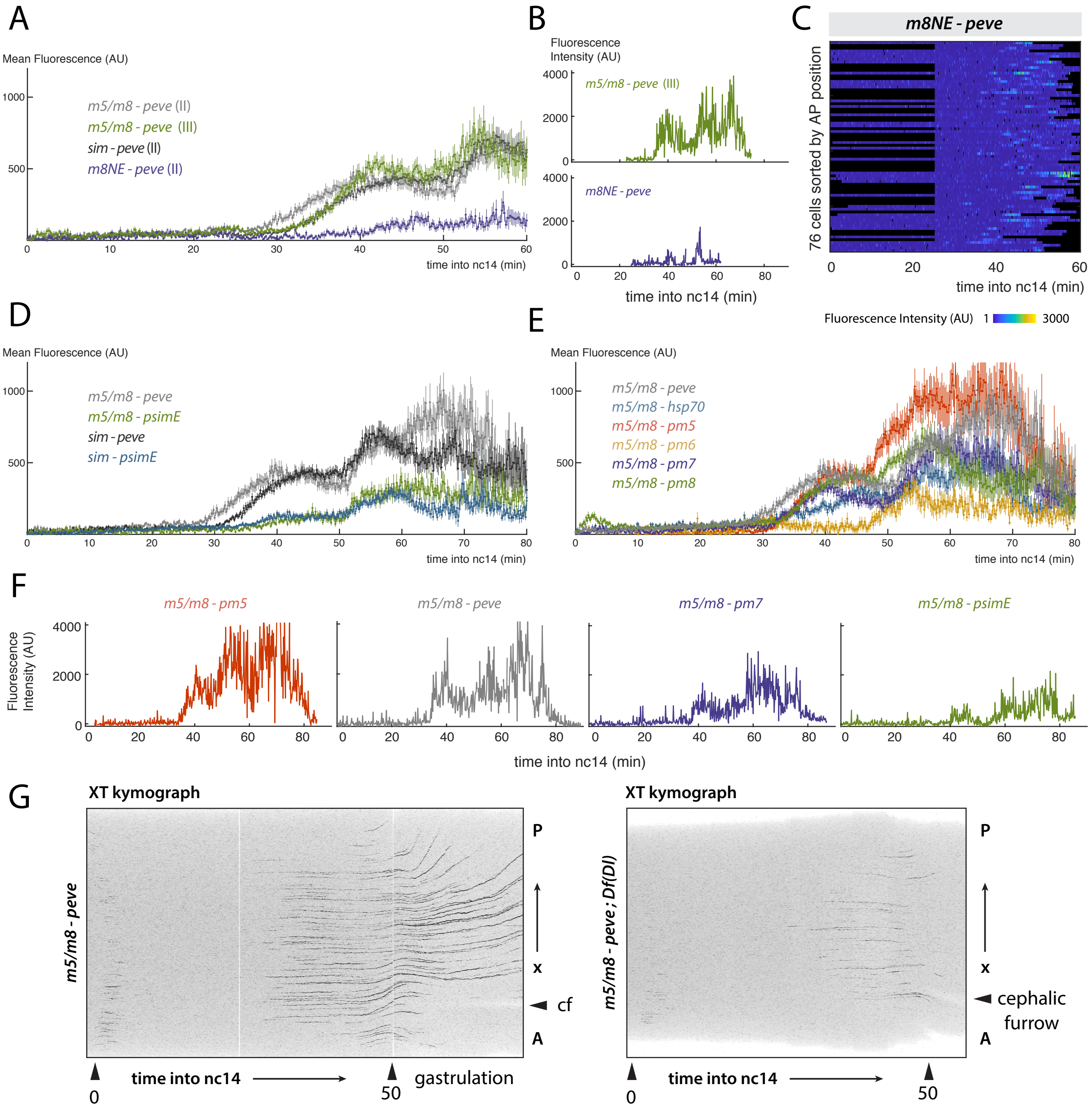

Figure S1. Related to Figure 1. The temporal profile of transcription is characteristic of MSE enhancers. **A**) Insertions of *m5/m8-peve* in landing sites in the second (light grey) and third (green) chromosome present the same temporal pattern and mean levels; and a Notch responsive neuroectodermal enhancer (*m8NE*, purple) presents a different temporal pattern than *m5/m8* and *sim*. **B**) Examples of traces from an *m5/m8-peve* insertion in a different genomic location, showing a continuous profile similar to Fig. 1D (top) and from *m8NE-peve*, a neuroectodemal enhancer that produces 'bursty' transcription in the MSE. **C**) *m8NE* produces asynchronized transcription in the MSE. **D**) The early promoter of *sim* (*psimE*) produces similar, lower mean levels of transcription from *m5/m8* and *sim* compared to the *eve* promoter. **E**) Different promoters from *E(spl)* complex genes and *hsp70* also affect the mean levels of activity but not the global pattern of transcription. **F**) Examples of fluorescent traces from different promoters. All produce continuous traces of different levels. **G**) Projections of the raw MCP-GFP channel over the Y and Z axes creating an XT kymograph. Only a few cells initiate transcription in embryos lacking zygotic Dl protein (right) compared to wild type embryos (left) and it is extinguished earlier. Mean and SEM are shown in **A**, **D** and **E**. Grey lines are re-plotted from Figs. 1D and 1G for comparison. n = 3 (*m5/m8-peve*III), 2 (*m8NE-peve*), 2 (*m5/m8-psimE*), 4 (*sim-psimE*), 3 (*m5/m8-hsp70*), 3 (*m5/m8-pm5*), 3 (*m5/m8-pm6*), 3 (*m5/m8-pm7*), 4 (*m5/m8-pm8*).

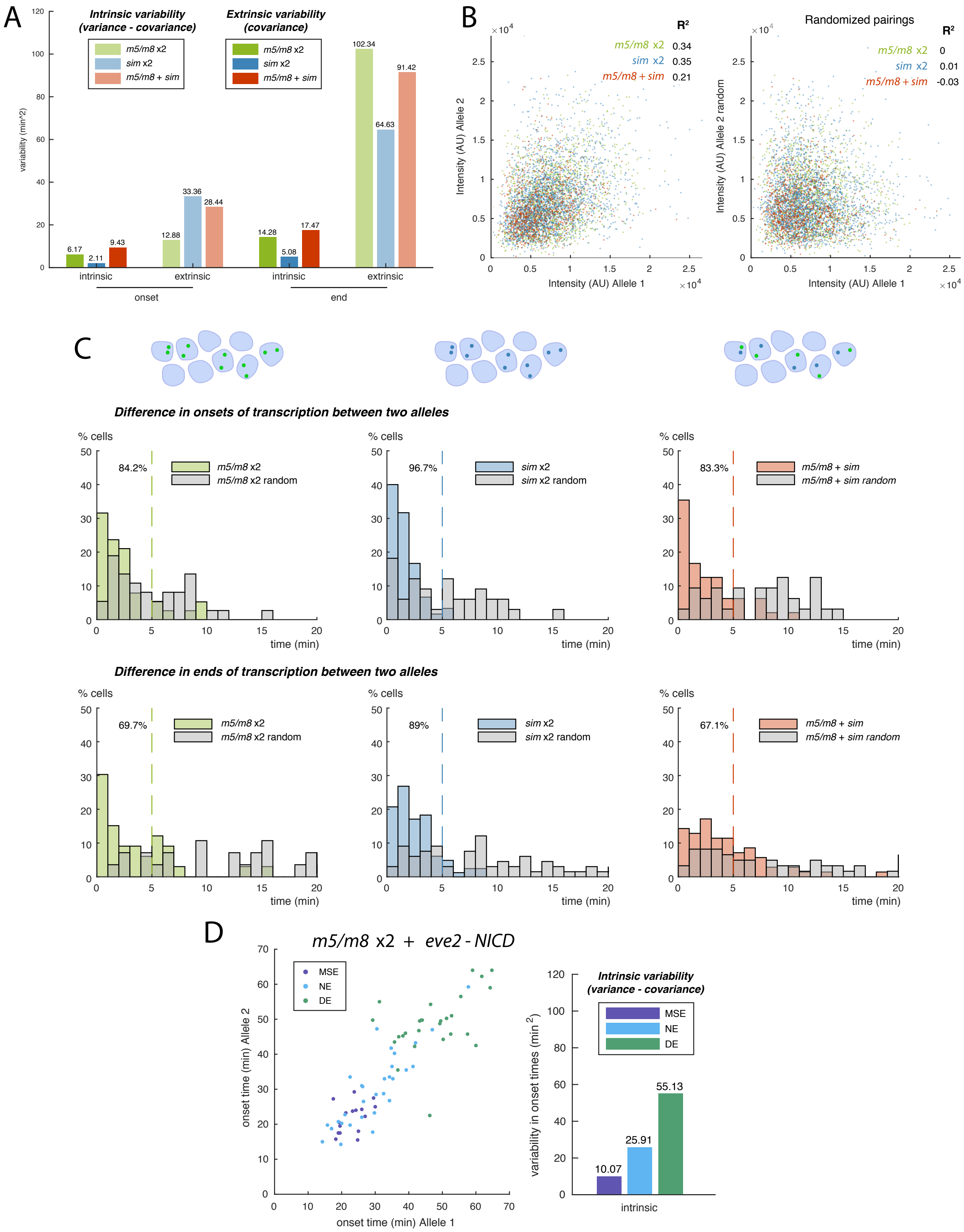

Figure S2. Related to Figures 2 and 3. Quantification of the variability intrinsic and extrinsic to transcription. A) Intrinsic (total variability minus covariance) and extrinsic (covariance) variability quantified in the onsets and ends of transcription using two MS2 reporters per cell. The amount of intrinsic variability is much smaller than the extrinsic and the intrinsic variability is higher in the ends than onsets of transcription for each combination. **B**) The fluorescence

Figure S2 (continued). intensities in two alleles at any timepoint present a small but significant correlation (left), compared to a correlation of 0 when the allele pairs are randomly assigned (right). Each color indicates the combination of 2 reporters compared. **C**) Histograms of the time difference between the appearance or dissapearance of transcription foci between the two reporters. The synchrony in the onset times is less than 5 min in more than 80% of the cells and more than 60% in the ends of transcription. Grey bars indicate the distribution of time differences when the allele pairs are randomly assigned. **D**) In conditions of ectopic Notch activity, nuclei in different regions present different intrinsic variability in the onset times of activation.

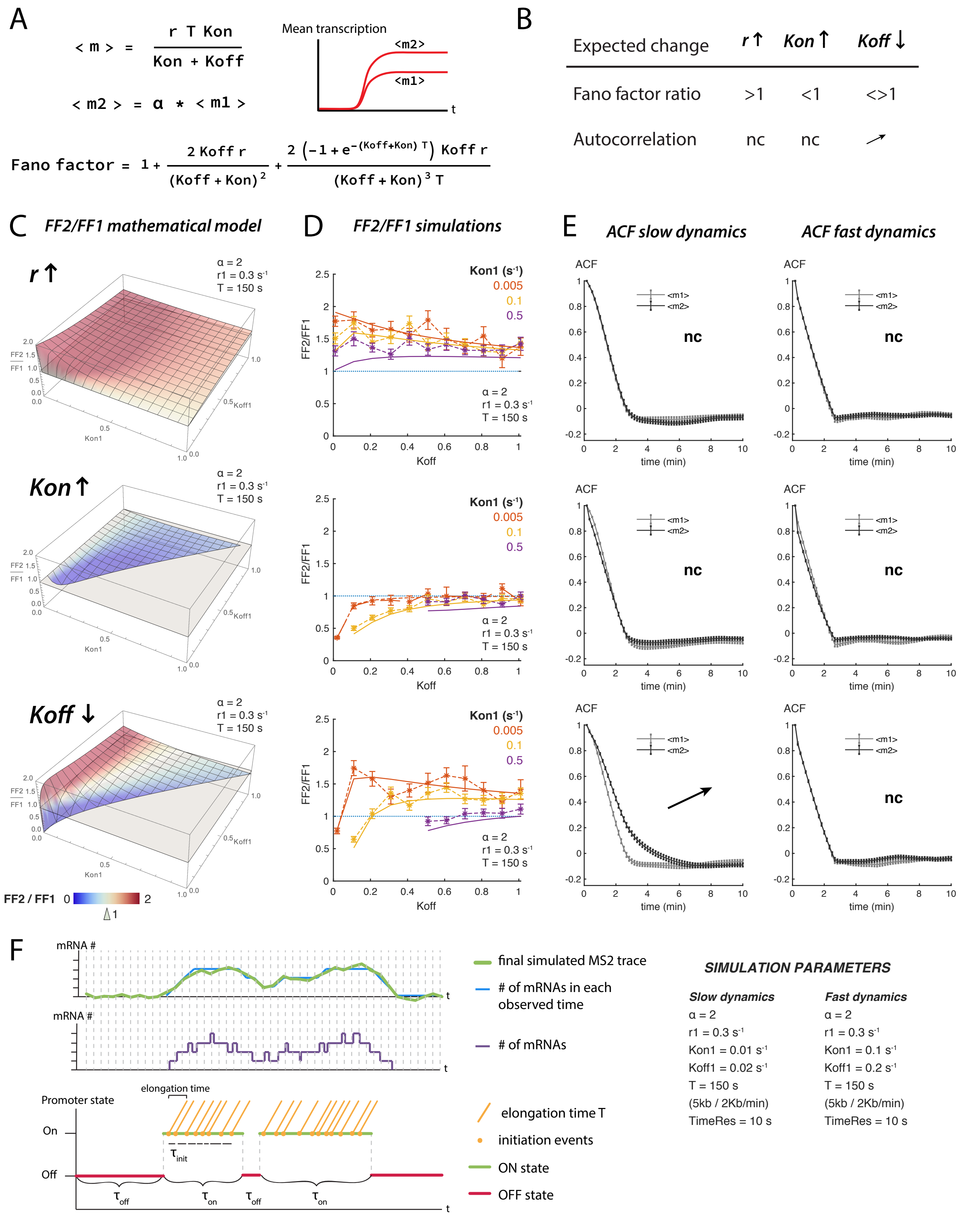

Figure S3. Related to Figure 4. Modelling a two-state promoter to infer changes in the kinetic parameters of transcription.

Figure S3 (continued). **E**) Expressions for the mean and Fano factor of the described 2-state model of transcription. Simulations and experiments compare the traces from two populations that have distinct means $\langle m1 \rangle$ and $\langle m2 \rangle$. α is the fold change in mean levels. The mean levels of transcription could increase from an increase in r, increase in K_{on} or decrease in K_{off}. **F**) Summary of the effects that modifying each parameter to produce an increase of α in the mean have on the Fano factor ratio (FFR = FF2/FF1) and autocorrelation function (ACF). When r increases, all FFR values are greater than 1 and no change (nc) in the ACF is observed. When K_{on} increases all FFR values are smaller than 1 and no change is observed in the ACF. When K_{off} decreases FFR values can be greater or smaller than 1 and the ACF presents a shift to the right when the dynamics are slow enough (see below). **G**) 3D plots representing the expected Fano factor ratio values from the mathematical model as a function of K_{on1} and K_{off1}. $\alpha = 2$, $r_1 = 0.3s^{-1}$ and $T = 150s$ in the three plots. The grey surface indicates FFR = 1. Only K_{on1} and K_{off1} values that produce allowed (ie. positive) K_{on2} and K_{off2} values are plotted (see Supplementary Methods for details). Surface map is colored based on FFR values ranging from 0 (blue) to 2 (red). When an increase of α in the mean is caused by an increase in r all FF ratio (FF2/FF1) values for any K_{on} and K_{off} values are greater than 1 (top plot). When it is due to an increase in K_{on} all FF ratios are smaller than 1 (middle plot). When K_{off} decreases to produce an increase of α in the mean, the obtained FF ratio values can be greater or smaller than 1 depending on the starting K_{on1} and K_{off1} parameters (bottom plot). **H**) Comparisons of the Fano factor ratios obtained from simulations of MS2 traces with different parameters (dashed lines) and the predicted from the mathematical model (solid line). Asterisks and error bars are mean and SD of the Fano factor ratio over 50 bootstraps of 1000 simulated MS2 traces, using the described K_{on1} and K_{off1} values and $\alpha = 2$, $r_1 = 0.3s^{-1}$, $T = 150s$ ($5Kb$ / $2Kb/min$). The expected trends in Fano factor ratios are correctly recovered in the simulations of transcription. **I**) Plots showing the changes ACF over time in simulated traces, comparing mean and SD of the ACF of 200 simulated MS2 traces in 50 bootstraps obtained from two groups: $\langle m1 \rangle$, grey, and $\langle m2 \rangle$, black. The parameters used for the simulations are $K_{on1} = 0.01$ and $K_{off1} = 0.02$ (slow dynamics, left column) or $K_{on1} = 0.1$ and $K_{off1} = 0.2$ (fast dynamics, right column) and $\alpha = 2$, $r_1 = 0.3s^{-1}$, $T = 150s$. No changes in the ACF are observed when the dynamics are fast. When the dynamics are slow, increases in r or K_{on} do not produce any change in the ACF but changes decreases in K_{off} shift the ACF to the right, from $\langle m1 \rangle$ to $\langle m2 \rangle$. **J**) Schematic representation of the steps to simulate MS2 traces. First ON and OFF states are generated based on the Gillespie algorithm, ON states are filled with initiation events that spread over their elongation time T. The final trace is obtained by counting the number of initiation events at each of the observed time points and adding gaussian noise to simulate experimental noise (see Supplementary Methods).

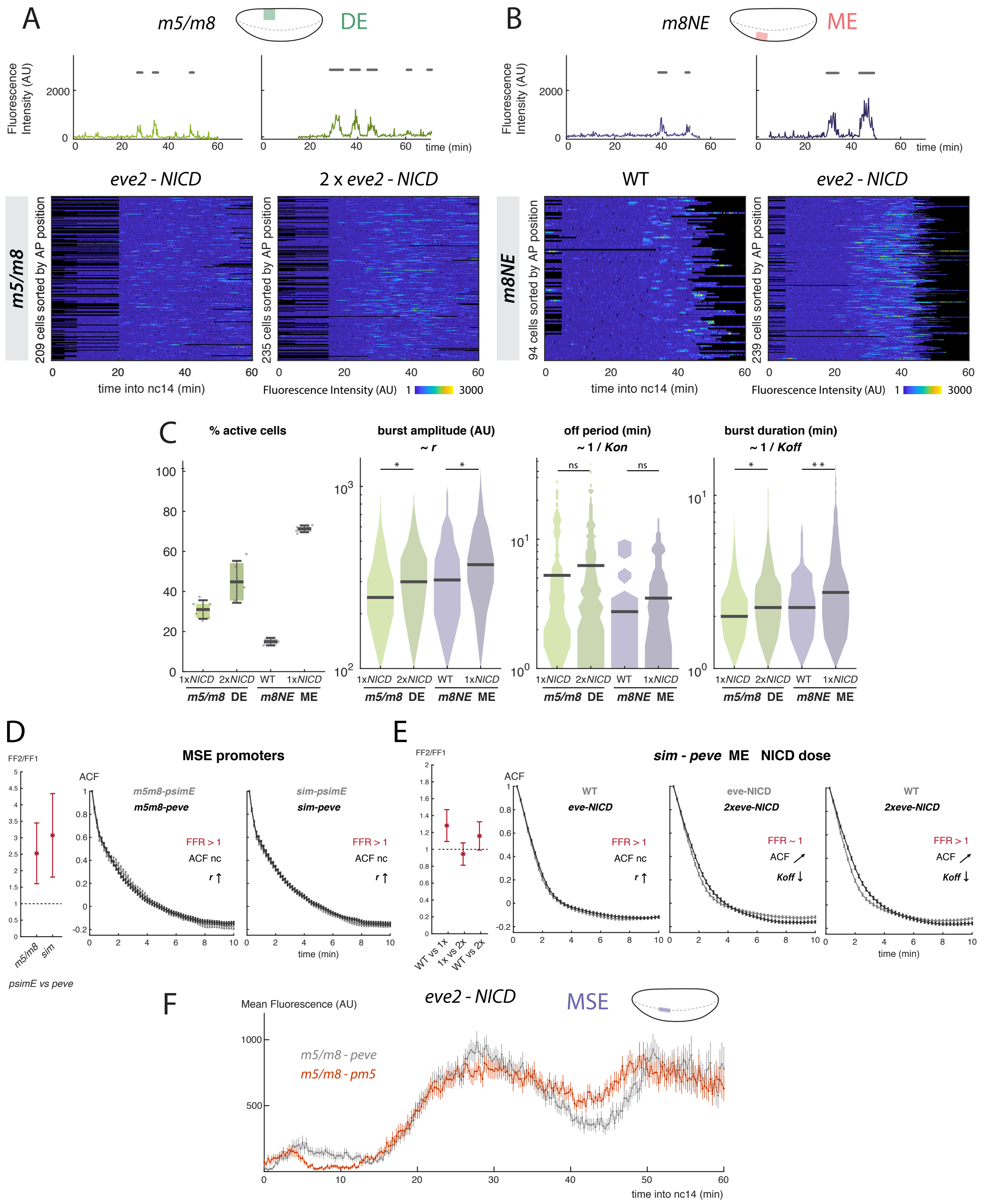

Figure S4. Related to Figure 4. Effects of NICD on the transcriptional bursting properties. A) Example traces and heatmaps of cells showing bursts of transcriptional activity from *m5/m8* in the dorsal ectoderm region in conditions of ectopic Notch activity. **B**) Example traces and heatmaps of cells showing bursts of transcriptional activity from *m8NE* in the mesoderm in wild type and *eve2-NICD* embryos. Burst periods are marked with a grey line. **C**) Quantification of the effects of NICD levels on the bursting properties. In both enhancers higher NICD produces a greater proportion of active cells and bigger bursts (increased amplitude and duration). **D-E**) Plots showing the Fano factor ratio and changes

Figure S4 (continued). in ACF over time (FFRatio in red, ACF in grey/black plots). FFRatio plots mean and SD of the FFRatio (FF2/FF1) in 50 bootstraps. Dashed line indicates 1 to compare the obtained FFRatio values. ACF plots compare mean and SD of the ACF of all available MS2 traces in 50 bootstraps from two conditions (grey and black lines as indicated, the mean levels are always higher in the condition plotted with a black line). **D**) Analysis of traces from reporters containing different promoters reveals changes in the mean are due to changes in r (FFRatio greater than 1 and no changes in the ACF). **E**) Comparison of the FF ratio and ACF in ME traces from *sim* in WT, *eve2-NICD* and 2x*eve2-NICD* reveals changes in the mean are consistent with increases in r (WT vs *eve2-NICD* comparison, left) or decreases in K_{off} (middle and right plots comparing *eve2-NICD* vs 2x*eve2-NICD* and WT vs *eve2-NICD*; ACF shifts to the right from the lower to higher mean condition). Note that the model assumes only one parameter changes. **F**) Higher NICD levels saturate the response from the effect on the enhancer. A promoter that produces higher mean levels in wild type embryos does not increase the levels with *eve2-NICD*. Differential distributions in **C** tested with two-sample Kolmogorov-Smirnov test: pvalues <0.01(*), $<10^{-5}$(**), $<10^{-10}$(***). n = 6 (*m5/m8 eve2-NICD* lateral view), 5 (*m5/m8* 2x*eve2-NICD* lateral view), 3 (*m8NE* WT), 5 (*m8NE eve2-NICD*) and 5 (*m5/m8-pm5 eve2-NICD*) embryos. Grey lines in **F** are re-plotted from Fig. 3H.

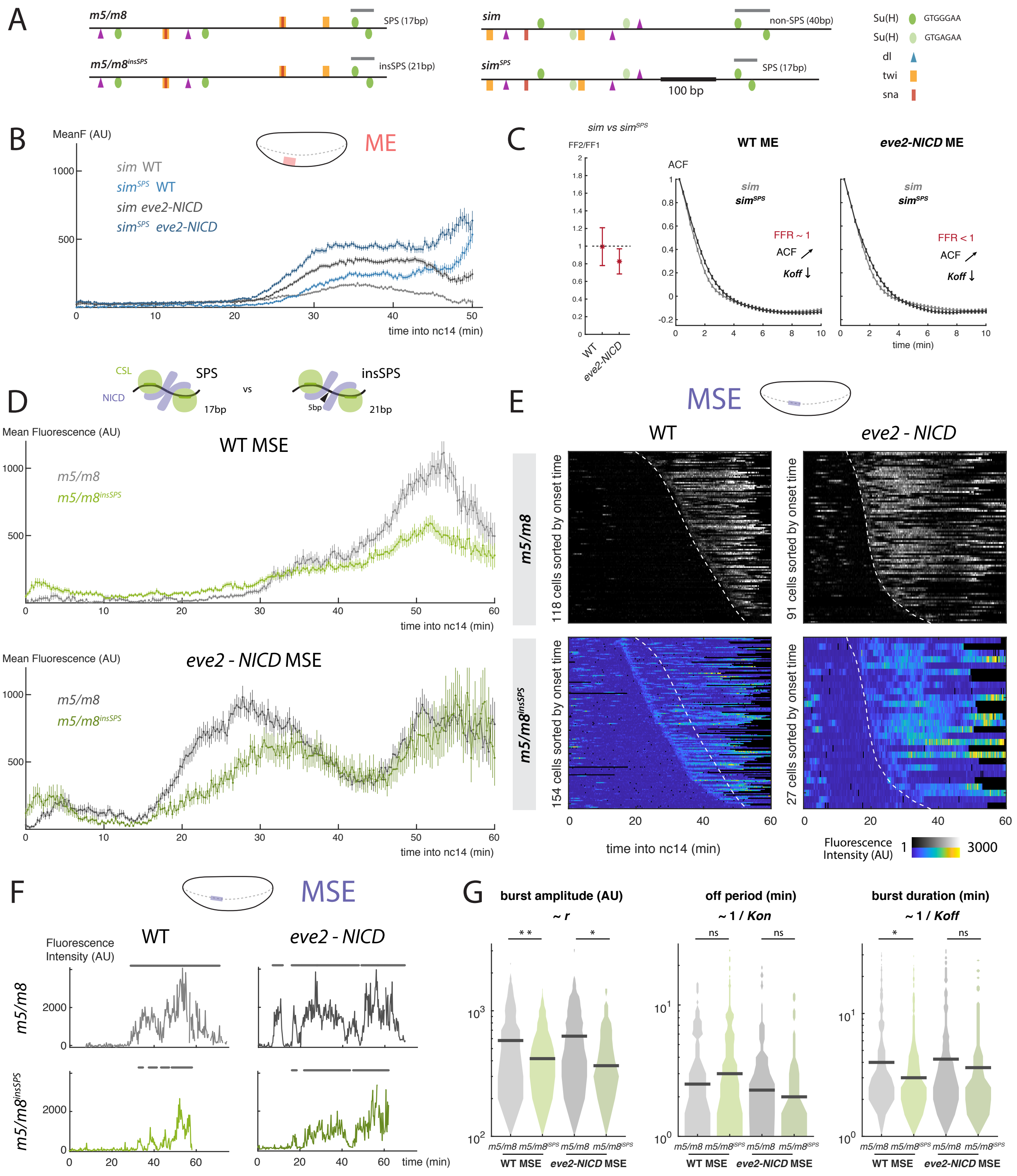

Figure S5. Related to Figure 5. Disruption of a SPS site produces lower transcription levels but does not delay the onset of transcription. A) Schematic representation of Su(H), Dorsal, Twist and Snail binding motifs in *m5/m8* and *sim* and introduced alterations in the SPS sites. **B**) sim^{SPS} produces higher mean levels in the mesoderm compared to *sim*, in both wild type and *eve2-NICD* embryos. **C**) Plots showing the Fano factor ratio and changes ACF over time (FFRatio in red, ACF in grey/black plots). The Fano factor ratio and autocorrelation function of *sim* and sim^{SPS} traces in the mesoderm in wild type and *eve2-NICD* embryos are compatible with changes in $Koff$ (shift in ACF) to produce increases in mean levels from *sim* to sim^{SPS}, in agreement with 5D. **D**) $m5/m8^{insSPS}$ produces lower mean levels of transcription compared to *m5/m8* but does not delay the onset of the response. **E**) $m5/m8^{ins}$ does not shift the onset of the response in *eve2-NICD* embryos (bottom) compared to *m5/m8* but presents some de-repression in wild type embryos (top). Dashed lines indicate onset times in the wild type enhancer. **F**) Examples of fluorescent traces in the mesectoderm region

Figure S5 (continued). in the described conditions. Burst periods are marked with a grey line. **G**) Quantification of the busting properties in the mesectoderm. $m5/m8^{insSPS}$ produces smaller bursts (lower amplitude and shorter duration) than *m5/m8*. Differential distributions in **G** tested with two-sample Kolmogorov-Smirnov test: pvalues <0.01(*), $<10^{-5}$(**), $<10^{-10}$(***). n = 5 ($m5/m8^{insSPS}$ WT), 3 ($m5/m8^{insSPS}$ *eve2-NICD*). Grey lines and heatmaps in **DE** are re-plotted from Fig. 3GH. **C** shows mean and SD over time of the mean Fano factor ratio and mean ACF over 50 bootstraps of all traces.

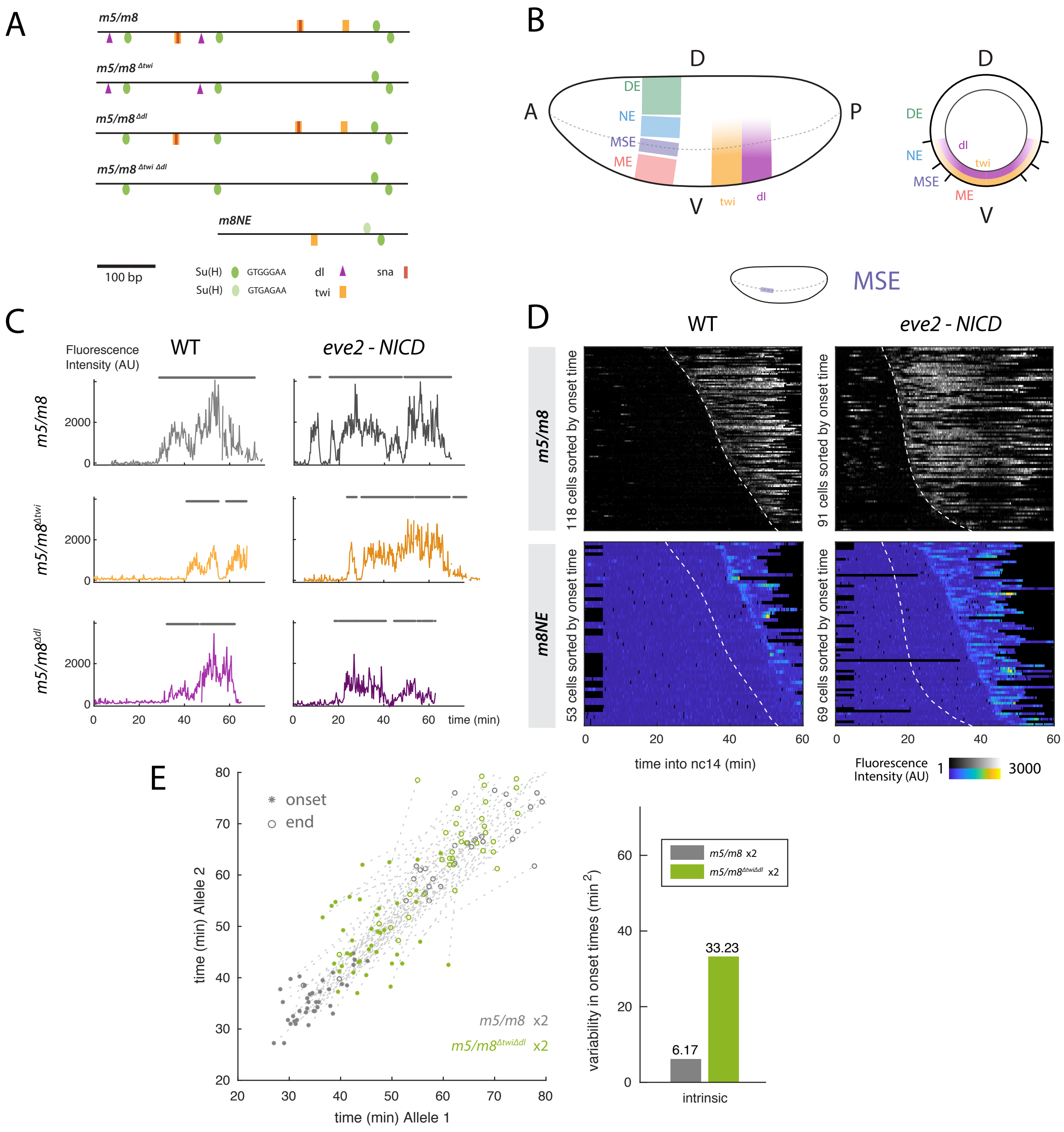

Figure S6. Related to Figure 6. Effects of mutations in Twist or Dorsal motifs in the onset of transcription. **A**) Schematic representation of the introduced mutations in *m5/m8* and comparison with a neuroectodermal enhancer, *m8NE*. **B**) Diagram of Twist and Dorsal gradients in the blastoderm embryo, showing lateral view (left) and cross-section (right). Both gradients extend in a ventral to dorsal gradient in the ME, MSE and NE. **C**) Examples of transcription traces from mesectodermal cells expressing *m5/m8* with mutated Twist or Dorsal motifs. The onset of transcription is delayed but transcription still occurs in a sustained manner. **D**) Heatmaps of MSE cells expressing *m8NE*. The onset of transcription is delayed compared to *m5/m8*. Dashed lines indicate onset times in the *m5/m8*. **E**) Quantification of the intrinsic variability in the transcription from a *m5/m8* enhancer with mutated Twist and Dorsal sites. Onset and end times for two $m5/m8^{\Delta twi \Delta dl}$-*peve* reporters in the same cell are shown and compared to *m5/m8-peve* (left). The intrinsic variability in the onset times of $m5/m8^{\Delta twi \Delta dl}$-*peve* increases compared to *m5/m8*. Grey dots and bar are re-plotted from Figures 2B and S2**A** for comparison. Greyscale heatmaps are duplicated from Fig. 3G.

Table S1. Related to STAR Methods. Primers used to amplify enhancer and promoter sequences and to introduce mutations in the enhancers. Restriction sites for *Hind*III, *Age*I and *Eag*I are underlined.

Primer name	Sequence
m5/m8 S	AAGCTTTGTTCCGTTTGGTAAAACCC
m5/m8 AS	ACCGGTCTTTCCACTGACATTCGAATC
sim S	AAGCTTCCCCGGCATATGTTACGCAC
sim AS	ACCGGTGGTTACAGGCAAACAGCAAAC
m8NE S	AAGCTTGGATCCCCTGCCCCTGCTC
m8NE AS	ACCGGTAACTTCGTAGGACGGAGGAC
peve S	AATGTCAGTGGAAAGACCGGTTTGCCTGCAGAGCGCAGCG
peve AS	TCCAAGGGCGAATTCACCGGCCGAACGAAGGCAGTTAGTTGTTGACTGT
hsp70 S	AATGTCAGTGGAAAGACCGGTGAGCGCCGGAGTATAAATAGA
hsp70 AS	TCCAAGGGCGAATTCACCGGCCGTATTCAGAGTTCTCTTCTTGTATTC
pm5 S	AATGTCAGTGGAAAGACCGGTACGCACGCACAGCATAGCAAT
pm5 AS	TCCAAGGGCGAATTCACCGGCCGAAGATTTGTAGAAATGTGCTGAGCTG
pm6 S	AATGTCAGTGGAAAGACCGGTTGGGATGATGTTGCTGCTG
pm6 AS	TCCAAGGGCGAATTCACCGGCCGTGTAGTATCACTTTACAGATAAGAGT
pm7 S	AATGTCAGTGGAAAGACCGGTAGTTTGCTCCGCAGGTGGT
pm7 AS	TCCAAGGGCGAATTCACCGGCCGATCTTTCGGAGGAGGTTATCCTG
pm8 S	AATGTCAGTGGAAAGACCGGTGCAGCTGTTCCTTGTGAAAAA
pm8 AS	TCCAAGGGCGAATTCACCGGCCGTTTGAAAAATTTTGTATTCGGCT
psimE S	AATGTCAGTGGAAAGACCGGTGTGTGAGTGTGGTGCATATAAATTTCGC
psimE AS	TCCAAGGGCGAATTCACCGGCCGGCGCACTCGCCGATGGTTAGTCA
sim for simSPS S	AAGTGTTTCCCACGATTCTGTCCTCCTTATGTGAAACTC
sim for simSPS AS	TCAAGTTTCCCACAAGATGGAAAGTGGAGAGTCCATAA
SPS from m5/m8 S	ATGGACTCTCCACTTTCCATCTTGTGGGAAACTTGAGG
SPS from m5/m8 AS	TTTCACATAAGGAGGACAGAATCGTGGGAAACACTTT
insSPS S	TGAGGGCAAAGAGGGGTGTTTCCCACGATTCGAAT
insSPS AS	TGGGAAACACCCCTCTTTGCCCTCAAGTTTCCCAC
mut Twi 1 S	ACTGATTTCCGTCCCAATGAGTCCCAAAATTGCACACATC
mut Twi 1 AS	TTTGGGACTCATTGGGACGGAAATCAGTATCTTACGGATT
mut Twi 2 S	CAAAATTCCCATTAGGACATCATCGGTTTGGCCCACTGTG
mut Twi 2 AS	AACCGATGATGTCCTAATGGGAATTTTGAGGGTGCCTTGC
mut Twi 3 S	CGGGACTCGCATTCGGACAACCTCCGATTATAACTTATAA
mut Twi 3 AS	ATCGGAGGTTGTCCGAATGCGAGTCCCGAGTCCGAGCTCC
mut dl 1 S	CCGTTTGGTGAGATCTCAAAAATCACATTCGAAAA
mut dl 1 AS	TGATTTTTGAGATCTCACCAAACGGAACAAAGCTT
mut dl 2 S	TCGCCTTGGGAGATCTCATTTCCGACATCCCAAAA
mut dl 2 AS	TCGGAAATGAGATCTCCCAAGGCGAAGATGTGTGC
